# Supplementary material for: Theory of correlated insulating behaviour and spin-triplet superconductivity in twisted double bilayer graphene
Source: Nat Commun. 2019 Nov 25;10:5333. doi: 10.1038/s41467-019-12981-1 (PMC6877569; doi:10.1038/s41467-019-12981-1)
Supplement: Supplementary file 1 — Supplementary Information [file 41467_2019_12981_MOESM1_ESM.pdf]

Supplementary Information

Theory of correlated insulating behaviour and spin-triplet superconductivity in twisted double bilayer graphene

Lee and Khalaf et al.

Supplementary Information

Supplementary Note 1. Details of Twisted Double Bilayer Graphene Continuum Model

Before getting into the detailed calculation, here we clarify some subtleties in the Moiré continuum approach and tight-binding parameters. As we will see, this is crucial because it can affect the physical band structures of a twisted double bilayer graphene. First, let us fix the lattice convention. Let $a = 1.42$ Å be the distance between carbon atoms. The original hexagonal lattice is defined by

$$a_1 = a(\sqrt{3}, 0), \quad a_2 = a(-\frac{\sqrt{3}}{2}, \frac{3}{2}) \quad \Leftrightarrow \quad G_1 = \frac{4\pi}{3a}(\frac{\sqrt{3}}{2}, \frac{1}{2}), \quad G_2 = \frac{4\pi}{3a}(0, 1), \quad G_3 = G_2 - G_1 \tag{1}$$

Here, $\mathbf{K}_\pm$-point is given by $\frac{4\pi}{3\sqrt{3}a}(\pm 1, 0)$. Once we twist two layers, Moiré structure is formed with a spatial modulation given by linear combinations of $\{\mathcal{R}_{\theta/2}G_1, \mathcal{R}_{\theta/2}G_2, \mathcal{R}_{-\theta/2}G_1, \mathcal{R}_{-\theta/2}G_2\}$, where $\mathcal{R}_\theta$ rotates a vector by angle θ counterclockwise. Periodicity of the given system is then governed by the smallest reciprocal lattice vector that can be obtained by linear combination. These are $(\mathcal{R}_{\theta/2} - \mathcal{R}_{-\theta/2})G_1$ and $(\mathcal{R}_{\theta/2} - \mathcal{R}_{-\theta/2})G_2$, which correspond to Moiré reciprocal lattice vectors. Therefore, we obtain $G^{\mathrm{M}}_{1,2} = \frac{8\pi \sin\theta/2}{3a}(-1/2, \pm\sqrt{3}/2)$. For convenience, use the following Moiré reciprocal lattice vectors from now on:

$$G^{\mathrm{M}}_1 = \frac{8\pi \sin\frac{\theta}{2}}{3a}\left(\frac{1}{2}, \frac{\sqrt{3}}{2}\right), \quad G^{\mathrm{M}}_2 = \frac{8\pi \sin\frac{\theta}{2}}{3a}\left(-\frac{1}{2}, \frac{\sqrt{3}}{2}\right), \quad a^{\mathrm{M}}_1 = \frac{\sqrt{3}a}{2\sin\frac{\theta}{2}}\left(\frac{\sqrt{3}}{2}, \frac{1}{2}\right), \quad a^{\mathrm{M}}_2 = \frac{\sqrt{3}a}{2\sin\frac{\theta}{2}}\left(-s\frac{\sqrt{3}}{2}, \frac{1}{2}\right) \tag{2}$$

Before getting into detail, let us fix the Fourier transform convention by $c^\dagger_{\mathbf{k}} = \sum_{\boldsymbol{R}} e^{i\mathbf{k}\cdot\boldsymbol{R}} c^\dagger_{\boldsymbol{R}}$. Here, $c^\dagger_{\boldsymbol{R}}$ creates a Wannier orbital $W(\boldsymbol{r} - \boldsymbol{R})$ centered at $\boldsymbol{R}$. This is consistent with the other convention used throughout the paper, where $|\psi_{\mathbf{k}}\rangle = \sum_R e^{i\mathbf{k}\cdot\boldsymbol{R}}|\boldsymbol{R}\rangle$. Under this choice, whenever there is a hopping from $\boldsymbol{R}$ to $\boldsymbol{R}'$ in real space, one obtains the term proportional to $e^{-i\mathbf{k}\cdot(\boldsymbol{R}'-\boldsymbol{R})}$ in the Bloch Hamiltonian. Here, the tight-binding model for bilayer graphene can be fully characterized by the parameters $(\gamma_0, \gamma_1, \gamma_3, \gamma_4, \Delta)$, where the hopping term for nearest neighbor γ_0 is intentionally taken to have an additional minus sign from the hopping integral so that all γ_i's are positive. The sign difference between vertical ($V_{pp\pi}$) and horizontal hopping ($V_{pp\sigma}$) overlap integral is originated from the phase structure of $2p_z$ orbital. (See how Slater-Koster parameters [1] are calculated) In Ref. [2], the sign of the trigonal warping γ_3 is taken to be negative, which is an inaccurate choice of the parameter because different sign convention would flip the shape of trigonal warping. Following the DFT result in Ref. [3], positive sign in front of γ_3 should be a proper choice for realistic materials.

Under this convention, where the phase structure for $2p_z$ orbitals at every carbon site is taken to be equivalent, one can derive the Moiré hopping term as the following. Once we have a Moiré structure, between momentum points of top and bottom layers, there exists Moiré-hopping term whose momentum transfer is given by linear combinations of G^{M}_1 and G^{M}_2. As we are interested in a Moire band structure near charge neutral point, we only consider electron momenta near Dirac points for top and bottom layers, $\mathcal{R}_{\pm\theta/2}\mathbf{K}_\pm$-points. For example, with respect to $\mathcal{R}_{\pm\theta/2}\mathbf{K}_+$, the momentum transfer condition can be written as

$$k_{\text{top}} - k_{\text{bot}} \equiv 0 \mod G^{\mathrm{M}}_{1,2} \qquad \Rightarrow \qquad (k_{\text{top}} - \mathcal{R}_{\theta/2}\mathbf{K}_+) - (k_{\text{bot}} - \mathcal{R}_{-\theta/2}\mathbf{K}_+) \equiv q_1 \mod G^{\mathrm{M}}_{1,2}, \tag{3}$$

where $q_1 = (\mathcal{R}_{-\theta/2} - \mathcal{R}_{\theta/2})\mathbf{K}_+ = K_M(0, -1)$ with $K_M = \frac{8\pi\sin\frac{\theta}{2}}{3\sqrt{3}a}$. Let us denote $\mathbf{K}^{\mathrm{t}}_+ = (\mathcal{R}_{\theta/2}\mathbf{K}_+)$ and $\mathbf{K}^{\mathrm{b}}_+ = (\mathcal{R}_{-\theta/2}\mathbf{K}_+)$. The Moiré state with a momentum p is given by a superposition of Bloch states of top and bottom layers with (absolute) momenta $\{p + n_1 G^{\mathrm{M}}_1 + n_2 G^{\mathrm{M}}_2 | n_1, n_2 \in \mathbb{Z}\}$. Since we want to solve the equation in terms of Dirac Hamiltonians with respects to $\mathbf{K}^{b,t}_+$-points, we do the following precedure. Define $\tilde{p} = p - \mathbf{K}^{\mathrm{t}}_+$. Then, the Moiré state with momenta p is composed of the Bloch states with the following momenta defined with respect to $\mathbf{K}^{\mathrm{t}}_+$ and $\mathbf{K}^{\mathrm{b}}_+$:

$$\begin{aligned} \text{Top Layer} : \{\tilde{p}, \tilde{p} - G^{\mathrm{M}}_1, \tilde{p} - G^{\mathrm{M}}_2, \tilde{p} - G^{\mathrm{M}}_1 + G^{\mathrm{M}}_2, \dots\} \quad &\text{with respect to } \mathbf{K}^{\mathrm{t}}_+ \\ \text{Bottom Layer} : \{\tilde{p} - q_1, \tilde{p} - q_2, \tilde{p} - q_3, \tilde{p} - q_2 + G^{\mathrm{M}}_2, \dots\} \quad &\text{with respect to } \mathbf{K}^{\mathrm{b}}_+ \end{aligned} \tag{4}$$

where $q_2 = (G^{\mathrm{M}}_1 + q_1) = K_M(\sqrt{3}/2, 1/2)$ and $q_3 = (G^{\mathrm{M}}_2 + q_1) = K_M(-\sqrt{3}/2, 1/2)$. Therefore, the state with a momentum $p = \tilde{p} + \mathbf{K}^{\mathrm{t}}_+$ can be solved by considering coupling among bloch states from $H(\mathcal{R}_{-\theta/2}[\tilde{p} - K^{\mathrm{t}}_{\mathrm{lat}}])$ and $H(\mathcal{R}_{\theta/2}[\tilde{p} - K^{\mathrm{b}}_{\mathrm{lat}}])$. Here, $K^{\mathrm{t,b}}_{\mathrm{lat}}$ is a vector denoting the location of each lattice point in $\{n_1 G^{\mathrm{M}}_1 + n_2 G^{\mathrm{M}}_2 : n_{1,2} \in \mathbb{Z}\}$ for the top layer and $\{q_1 + n_1 G^{\mathrm{M}}_1 + n_2 G^{\mathrm{M}}_2 : n_{1,2} \in \mathbb{Z}\}$ for the bottom layer in the k-space.

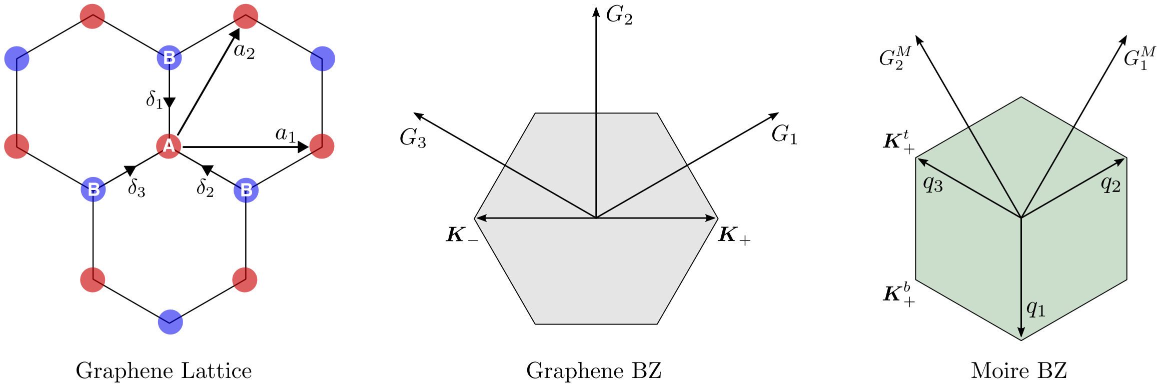

Supplementary Figure 1. Real space lattice and Brillouin zone for a single graphene layer, and Brillouin zone orignated from $\boldsymbol{K}_+$ valley for a twisted (double) bilayer graphene. The figure illustrates all vectors labeled for the discussion in the manuscript.

Let top and bottom layer have relative shift δ. For convenience, take a frame where the bottom layer is fixed and the top layer is rotated by θ. Then, Each original Bloch state is represented as

$$\left|\psi^{\mathrm{t}}_{p,\beta}\right\rangle = \frac{1}{\sqrt{N}}\sum_{R} e^{ip\cdot(R'+\tau'_\beta)}\left|R'+\tau'_\beta\right\rangle, \quad \left|\psi^{\mathrm{b}}_{k,\alpha}\right\rangle = \frac{1}{\sqrt{N}}\sum_{R'} e^{ik\cdot(R+\tau_\alpha)}\left|R+\tau_\alpha\right\rangle \tag{5}$$

where α,β are sublattice indices and τ_α is associated displacement. Here, primed coordinate $R'=\mathcal{R}_\theta(R+\delta)$ and $\tau'=\mathcal{R}_\theta\tau$ are for the top layer, meaning that it is rotated by θ along counter-clockwise direction. Here, $R=n_1a_1+n_2a_2$. The initial displacement δ between layers is not important in the end, as we will see. Let $\tau_0=a(0,1)$. In the mono-mono case $\delta=0$ for $A-A$ stacking and $\delta=\tau_0$ for $A-B$ stacking. $A-B$ stacking means that A-site of the top layer is placed at the location of B-site of the bottom layer.

By definition, A-site is original site spanning the lattice, and B-site is displaced by τ_0 with respect to the A-site. In the bilayer(AB)-bilayer(AB) case, $\delta=-\tau_0$ because between layers it is BA stacking. For the following calculation, we take $\delta=-\tau_0$ and $d=0$. Now, hopping matrix element from top second layer to bottom first layer $H_{b,t}$ can be evaluated by

$$\begin{aligned}
T^{\alpha\beta}_{kp'} &= \left\langle\psi^{\mathrm{b}}_{k\alpha}\right| H_T \left|\psi^{\mathrm{t}}_{p'\beta}\right\rangle = \frac{1}{N}\sum_{R,R'} e^{-ik(R+\tau_\alpha)+ip(R'+\tau'_\beta)}\left\langle R+\tau_\alpha\right| H_T \left|R'+\tau'_\beta\right\rangle \\
&= \left\langle\psi^{\mathrm{b}}_{k\alpha}\right| H_T \left|\psi^{\mathrm{t}}_{p'\beta}\right\rangle = \frac{1}{N}\sum_{R,R'} e^{-ik(R+\tau_\alpha)+ip(R'+\tau'_\beta)}\cdot t(R+\tau_\alpha-R'-\tau_\beta) \\
&= \frac{1}{N}\sum_{R,R'} e^{-ik(R+\tau_\alpha)+ip(R'+\tau'_\beta)}\frac{1}{\Omega}\int d^2q\, t_q e^{iq\cdot(R+\tau_\alpha-R'-\tau'_\beta)} \\
&= \frac{1}{N\Omega}\int d^2q \sum_{R,R'} t_q e^{-i(k-q)\cdot(R+\tau_\alpha)+i(p-q)(R'+\tau'_\beta)} \\
&= \sum_{q\in\mathrm{BZ}} t_q\, e^{-iG_1\cdot\tau_\alpha+iG'_2(\tau'_\beta+\delta')} \text{ non-zero only when } k-q=G_1\in\mathcal{G} \text{ and } p-q=G'_2\in\mathcal{G}' \\
&= \sum_{g_1,g_2} t_{k-g_1}\, e^{-ig_1\cdot\tau_\alpha+ig_2\cdot(\tau_\beta+\delta)}\cdot\delta_{p+g_1,k+g'_2} \\
&= \sum_{g_1,g_2} t_{k+g_1}\, e^{ig_1\cdot\tau_\alpha-ig_2\cdot(\tau_\beta+\delta)}\cdot\delta_{p-g_1,k-g'_2}
\end{aligned} \tag{6}$$

where we used $t(R)=\frac{1}{N}\sum_q e^{iq\cdot R}t_q \approx \frac{1}{\Omega}\int d^2q\, e^{iq\cdot R}t_q$, and $\mathcal{G}$ and $\mathcal{G}'$ are sets of reciprocal lattice vectors for original bottom and top lattices, respectively. In principle, for a different gauge choice $t(\boldsymbol{R})$ depends on α,β indices as well, but since we took the gauge choice where all phase structures for $2p_z$ orbitals are the same, the dependence will be trivial. In the last line, we just did change of variables. Now we can see that it is nonvanishing only when

$$p-k=g_1-\mathcal{R}_\theta g_2 \quad \text{for some reciprocal lattice vectors } g_1,g_2\in\mathcal{G} \tag{7}$$

Considering that t_q is decreasing fast with q, we can only retain most relevant terms where $k+g_1$ is minimized. In terms of a momentum relative to the $\mathbf{K}_+^{\mathrm{t,b}}$ points, we have

$$(p-\mathbf{K}_+^{\mathrm{t}})-(k-\mathbf{K}_+^{\mathrm{b}})=(g_1-\mathcal{R}_\theta g_2)+q_1. \tag{8}$$

Naively, when we consider $k+g_1$, since k does not deviate much from $\mathbf{K}_+^{\mathrm{b}}$-point, the most relevant t_{k+g_1} would be given when $g_1=g_2=0, G_3, -G_1$ so that $\left|\mathbf{K}_+^{\mathrm{b}}+g_1\right|=\left|\mathbf{K}_+^{\mathrm{b}}\right|$. For cases with $g_1\neq g_2$, (i) the energy difference between top and bottom electrons are very large, and (ii) t_{k+g_1} would be small, and therefore we ignore such cases. In fact, each of these cases corresponds to when $\tilde{p}-\tilde{k}=q_1,q_2,q_3$:

$$\begin{aligned} g_1=0 &\longrightarrow \tilde{p}-\tilde{k}=q_1\\ g_1=G_3 &\longrightarrow \tilde{p}-\tilde{k}=q_1+G_3-\mathcal{R}_\theta G_3=q_1+G_1^{\mathrm{M}}=q_2\\ g_1=-G_1 &\longrightarrow \tilde{p}-\tilde{k}=q_1-G_1+\mathcal{R}_\theta G_1=q_1+G_2^{\mathrm{M}}=q_3. \end{aligned} \tag{9}$$

With this understanding, one can write down three hopping matrices as the following:

$$T(q_1)=\begin{pmatrix}1&1\\1&1\end{pmatrix}\quad T(q_2)=\begin{pmatrix}z&1\\z^*&z\end{pmatrix}=e^{-iG_3\cdot\delta}\begin{pmatrix}1&z^*\\z&1\end{pmatrix}\quad T(q_3)=\begin{pmatrix}z^*&1\\z&z^*\end{pmatrix}=e^{iG_1\cdot\delta}\begin{pmatrix}1&z\\z^*&1\end{pmatrix} \tag{10}$$

where $z=e^{2\pi i/3}$ and since $G_3\cdot\tau_0=G_1\cdot\tau_0=2\pi/3$. Due to the fact that $\delta=-\tau_0$ instead of τ_0, the form is slightly different from the TBG case [4, 5]. By proper phase redefinition of Bloch states represented by momentum lattices (gauge degrees of freedom for Bloch states), we can absorb z and z^* factors in front of matrices. Thus, the form of the hopping matrices can be simplified. Moreover, an initial displacement between two layers is not important. In this derivation, it is not difficult to notice that diagonal and off-diagonal entries for Moiré hopping matrices can be different. If there is an additional spatial modulation with a Moiré scale, given differently between AA(BB) and AB(BA) sites in $t(R+\tau_\alpha-R'-\tau_\beta)$, one would obtain a different values for t_k, as explained in Ref. [6, 7]. Finally, to obtain an energy spectrum at Moiré momentum k, one needs to diagonalize the following Hamiltonian with a certain cutoff:

$$H=\begin{pmatrix}H_t(k)&T^\dagger(q_i)&...\\T(q_i)&H_b(k+q_i)&...\\...&...&...\end{pmatrix} \tag{11}$$

Now, we want to point out some subtlety for the generic Moiré hopping matrix:

$$T_n=w_0+w_1e^{2\pi n\sigma_3/3}\sigma_1e^{-2\pi n\sigma_3/3} \tag{12}$$

where the Pauli operator σ_i acts on the sublattice basis. This is the form of the hopping term written in Ref. [4], where the $\mathbf{K}$ and $\mathbf{K}'$ Dirac Hamiltonian was written as[1]

$$H(\mathbf{K}+\mathbf{k})=\hbar v_{\mathrm{F}}\begin{pmatrix}0&k_x-ik_y\\k_x+ik_y&0\end{pmatrix}\qquad H(\mathbf{K}'+\mathbf{k})=-\hbar v_{\mathrm{F}}\begin{pmatrix}0&k_x+ik_y\\k_x-ik_y&0\end{pmatrix}. \tag{13}$$

Now, imagine we choose a different basis choice, for example multiplying $(-)$ sign for the B-sublattices. This is equivalent to apply σ_3 transformation to the operators, and as a result, both $H(\mathbf{K}+\mathbf{k})$ and $H(\mathbf{K}'+\mathbf{k}')$ would change its sign. This is the basis chosen in Ref. [5]. Accordingly, interlayer hopping term T_0 would change as well, from $w_0+w_1\sigma_1$ to $w_0-w_1\sigma_1$ and similarly for others.

[1] $H(\mathbf{K}+\mathbf{k})$ in the MacDonald's paper is a Hamiltonian with respect to the $\mathbf{K}'$-point in our convention.

Supplementary Note 2. Supplementary Note 2. Interaction Projection and Intervalley Hund's Coupling

Here, we provide the details for the procedure of projecting the Coulomb interaction on the isolated flat band and how to derive the intervalley Hund's coupling. The interaction Hamiltonian can be written as

$$\mathcal{H}_{\text{int}} = \frac{1}{2}\int d\boldsymbol{r}_1 d\boldsymbol{r}_2 \rho(\boldsymbol{r}) V(\boldsymbol{r}-\boldsymbol{r}')\rho(\boldsymbol{r}'). \tag{14}$$

Here, $\boldsymbol{r}$ integration is over the whole space not just the unit cell. For the screed Coulomb interaction, $V(\boldsymbol{r})$ is given by

$$V(\boldsymbol{r}) = \frac{e^2}{4\pi\epsilon\epsilon_0}\frac{e^{-\kappa|\boldsymbol{r}|}}{|\boldsymbol{r}|}. \tag{15}$$

where κ denotes the inverse screening length. The density operator is given by

$$\hat{\rho}(\boldsymbol{r}) = \sum_{n,n',\sigma,\sigma',\tau,\tau'} c^\dagger_{n,\sigma,\tau}(\boldsymbol{r})c_{n',\sigma',\tau'}(\boldsymbol{r}), \tag{16}$$

where σ, σ' sum over spin states $\uparrow,\downarrow$ and τ, τ' sum over vallyes $\pm$, and n sums over the relevant set of bands. In the following, we will restrict ourselves to the isolated Moiré band and drop the band index n. Expansion in the Bloch basis is done by writing

$$c_{\sigma,\tau}(\boldsymbol{r}) = \frac{1}{\sqrt{N}}\sum_{\mathbf{k}\in\text{BZ}} \psi_{\sigma,\tau,\mathbf{k}}(\boldsymbol{r})c_{\sigma,\tau}(\mathbf{k}), \tag{17}$$

where N is the number of momentum point in the first Brillouin zone which equals to the total number of Moiré unit cells in system, and $\psi_{\mathbf{k}}(\boldsymbol{r})$ are the Bloch states satisfying $\psi_{\mathbf{k}}(\boldsymbol{r}+\boldsymbol{R}) = e^{i\mathbf{k}\cdot\boldsymbol{R}}\psi_{\mathbf{k}}(\boldsymbol{r})$ for a given Moiré lattice translation $\boldsymbol{R}$. We now split the density into intra- and intervalley components

$$\hat{\rho}(\boldsymbol{r}) = \sum_{\sigma,\tau}[\hat{\rho}^+_{\sigma,\tau}(\boldsymbol{r}) + \hat{\rho}^-_{\sigma,\tau}(\boldsymbol{r})], \tag{18}$$

$$\hat{\rho}^\pm_{\sigma,\tau}(\boldsymbol{r}) = \frac{1}{N}\sum_{\mathbf{k},\mathbf{k}'\in\text{BZ}} \psi^\dagger_{\tau,\mathbf{k}}(\boldsymbol{r})\psi_{\pm\tau,\mathbf{k}'}(\boldsymbol{r})c^\dagger_{\sigma,\tau}(\mathbf{k})c_{\sigma,\pm\tau}(\mathbf{k}'). \tag{19}$$

Here, we used the fact that different spin states are orthogonal. If valley symmetry is exact, states belonging to different valleys would also be orthogonal leading to a vanishing intervalley density $\rho^-_{\sigma,\tau}$. However, valley symmetry is broken on the scale of $|K-K'|^{-1}$ leading to a very small intervalley Hund's coupling term. This term can be usually neglected since it is much smaller than the interaction between intravalley densities. Nevertheless, contributions from this term can lift the degeneracy between different broken symmetry states which are otherwise exactly degenerate, which makes it important to include it in our analysis. We note that the Bloch states are generally vectors with some internal index denoting layer, sublattice, etc which means that the combination $\psi^\dagger\psi$ above denotes an inner product in these internal indices.

The Bloch states can be written in terms of the periodic function $u_{\mathbf{k}}(\boldsymbol{r})$ which can be expanded in a Fourier series in reciprocal lattice vectors $\boldsymbol{G}$ leading to

$$\psi_{\mathbf{k}}(\boldsymbol{r}) = e^{i\mathbf{k}\cdot\boldsymbol{r}}u_{\mathbf{k}}(\boldsymbol{r}) = \frac{1}{\sqrt{|\Omega|}}\sum_{\boldsymbol{G}} e^{i(\boldsymbol{G}+\mathbf{k})\cdot\boldsymbol{r}}u_{\mathbf{k}}(\boldsymbol{G}), \tag{20}$$

where $\boldsymbol{G}$ is the Moiré reciprocal lattice vector, and Ω is the area of the Moiré unit cell. Here, $u_{\mathbf{k}}(\boldsymbol{G})$ are normalized such that $\sum_{\boldsymbol{G}} u^\dagger_{\mathbf{k}}(\boldsymbol{G})u_{\mathbf{k}}(\boldsymbol{G}) = 1$. In addition, we can choose the gauge such that the Bloch states satisfy

$$u_{\mathbf{k}+\boldsymbol{G}_0}(\boldsymbol{G}) = u_{\mathbf{k}}(\boldsymbol{G}+\boldsymbol{G}_0). \tag{21}$$

If the band has a non-vanishing Chern number, it is impossible to choose a smooth and periodic gauge and there would be an additional phase factor in front of the RHS [8]. In this case, the condition (Eq. 21) implies a discontinuity of the phase of $u_{\mathbf{k}}$ at the Brillouin zone boundaries.

The interacting Hamiltonian in momentum space is given by

$$\mathcal{H}_{\text{int}} = \frac{1}{2\,\text{Vol}}\sum_{\boldsymbol{q}} \hat{\rho}(\boldsymbol{q})V(\boldsymbol{q})\hat{\rho}(-\boldsymbol{q}) \tag{22}$$

where $V(\boldsymbol{q}) = \int d\boldsymbol{r} V(\boldsymbol{r}) e^{-i\boldsymbol{q}\boldsymbol{r}}$ and Vol $= N\Omega$. We note that the Fourier transform of $\rho^{\pm}(\boldsymbol{r})$ is not restricted to momenta inside the Moiré BZ and it should be expressed in terms of a general momentum $\boldsymbol{q}$. The density $\hat{\rho}(\boldsymbol{q})$ is generally non-periodic in $\boldsymbol{q}$ under reciprocal Moiré lattice translations since the Bloch states have a non-trivial spatial structure inside the Moiré unit cell. Instead, it decays over some momentum scale comparable to the Moiré Brillouin zone size. On the other hand, the Bloch states has no structure inside the unit cell of the original bilayer graphene where a tight-binding description of the orbitals was employed. Hence, the density $\hat{\rho}(\boldsymbol{q})$ is periodic under any reciprocal lattice translation for the original system. As a result, $\hat{\rho}(\boldsymbol{q})$ consists of several identical narrow peaks centered at reciprocal lattice vectors of the original bilayer graphene $\tilde{\boldsymbol{G}}$ for the intravalley density ρ^{+} or at $K - K' + \tilde{\boldsymbol{G}}$ for the intervalley density ρ^{-}. This poses a problem since it implies that the summation over $\boldsymbol{q}$ in Eq. 22 diverges.

To resolve this issue, we notice that the periodicity of $\hat{\rho}(\boldsymbol{q})$ in reciprocal space for the original lattice is an artifact of the tight-binding approximation, where an atomic orbital is taken to be point-like. If we instead use the actual shape of the Wannier orbital, the density operator $\hat{\rho}(\boldsymbol{q})$ will decay for momenta larger than a certain cutoff Λ which is given by the inverse size of the Wannier orbitals. Rather than attempting to precisely determine the value of Λ from the graphene Wannier orbitals, we will consider Λ as a phenomenological parameter of the same order as the size of the original Brillouin zone. This will have the effect of restricting the sum over momenta in Eq. 22 to the vicinity of $\boldsymbol{q} = 0$ for the intravalley density $\hat{\rho}^{+}(\boldsymbol{q})$ and the vicinity of $K - K'$ and $R_{\pm 2\pi/3}(K - K')$ for the intervalley density $\hat{\rho}^{-}(\boldsymbol{q})$.

Therefore, we restrict ourselves to the vicinity of 0 for $\rho^{+}(\boldsymbol{q})$ and $K - K'$ (and its rotation related points) for $\rho^{-}(\boldsymbol{q})$. In the following, we perform Fourier transform in terms of small deviations around these momenta by defining $\rho^{\alpha}_{\sigma,\tau}(\boldsymbol{q})$ as(note that $c_{\mathbf{k}+\boldsymbol{G}} = c_{\mathbf{k}}$):

$$\rho^{\alpha}_{\sigma,\tau}(\boldsymbol{q}) \equiv \int_{N\Omega} d\boldsymbol{r} e^{-i[\boldsymbol{q} - \frac{1-\alpha}{2}(K_\tau - K_{\alpha\tau})]\cdot \boldsymbol{r}} \rho^{\alpha}(\boldsymbol{r}) = \sum_{\mathbf{k}} \lambda^{\alpha}_{\tau,\boldsymbol{q}}(\mathbf{k}) c^{\dagger}_{\sigma,\tau}(\mathbf{k}) c_{\sigma,\alpha\tau}(\mathbf{k}+\boldsymbol{q}), \qquad \alpha = \pm \tag{23}$$

Here, we introduced $K_{+} = K$ and $K_{-} = K'$ and we used that $\psi_{\tau,\mathbf{k}}(\boldsymbol{r}) = \sum_{\boldsymbol{G}} e^{i(\mathbf{k}+K_\tau+\boldsymbol{G})\cdot\boldsymbol{r}} u_{\tau,\mathbf{k}}(\boldsymbol{G})$. In addition, we introduced the intra- and intervalley form factors defined by

$$\begin{aligned} \lambda^{\pm}_{\tau,\boldsymbol{q}}(\mathbf{k}) &= \sum_{\mathbf{k},\mathbf{k}' \in \text{MBZ}} \sum_{\boldsymbol{G},\boldsymbol{G}'} \delta_{K_\tau+\mathbf{k}+\boldsymbol{G}+\boldsymbol{q}, K_{\pm\tau}+\mathbf{k}'+\boldsymbol{G}'} \cdot u^{\dagger}_{\tau,\mathbf{k}}(\boldsymbol{G}) u_{\pm\tau,\mathbf{k}'}(\boldsymbol{G}') \\ &= \sum_{\boldsymbol{G}} u^{\dagger}_{\tau,\mathbf{k}}(\boldsymbol{G}) u_{\pm\tau,\boldsymbol{p}(\mathbf{k}+\boldsymbol{q})}(\boldsymbol{G} + \boldsymbol{G}(\mathbf{k}+\boldsymbol{q})) \equiv \langle u_{\tau,\mathbf{k}} | u_{\pm\tau,\mathbf{k}+\boldsymbol{q}} \rangle \end{aligned} \tag{24}$$

The function $\boldsymbol{p}(\boldsymbol{q})$ and $\boldsymbol{G}(\boldsymbol{q})$ are defined to give the projection onto the first BZ and the reciprocal lattice vector corresponding to $\boldsymbol{q}$, respectively, such that $\boldsymbol{q} = \boldsymbol{G}(\boldsymbol{q}) + \boldsymbol{p}(\boldsymbol{q})$. The last equality is important for the numerical implementation because the summation over $\boldsymbol{q}$ can go outside the first BZ whereas the numerical calculation is only carried out in the first BZ.

Time-reversal symmetry dictates that

$$u_{\tau,\mathbf{k}}(\boldsymbol{G}) = u^{*}_{-\tau,-\mathbf{k}}(-\boldsymbol{G} + \boldsymbol{G}_0), \tag{25}$$

for some reciprocal lattice vector $\boldsymbol{G}_0$. This relation can be exploited for the evaluation of form factors. In fact, a direct numerical evaluation gives $\boldsymbol{G}_0$ in our setting.

The form factors satisfy the identities

$$\begin{aligned} [\lambda^{\pm}_{\tau,\boldsymbol{q}}(\mathbf{k})]^{*} = \lambda^{\pm}_{\pm\tau,-\boldsymbol{q}}(\mathbf{k}+\boldsymbol{q}), \quad \lambda^{\pm}_{\tau,\boldsymbol{q}}(\mathbf{k}) = [\lambda^{\pm}_{-\tau,-\boldsymbol{q}}(-\mathbf{k})]^{*}, \\ \lambda^{\pm}_{\tau,\boldsymbol{q}}(\mathbf{k}+\boldsymbol{G}) = \lambda^{\pm}_{\tau,\boldsymbol{q}}(\mathbf{k}). \end{aligned} \tag{26}$$

The first identity follows from the definition of the form factor, the second from time-reversal symmetry (Eq. 25) and the third from our periodic gauge choice (Eq. 21).

Finally, the resulting interaction can be expanded as a sum of four terms: $\rho^{+}\rho^{+}$ containing intravalley densities, $\rho^{-}\rho^{-}$ containing intervalley densities and two cross terms $\rho^{+}\rho^{-}$. The latter ones have to vanish since they necessarily involve densities at large momenta $\boldsymbol{q} \pm (K_{+} - K_{-})$ (due to the factor $\lambda_{\boldsymbol{q}}$ which is assumed to decay with $\boldsymbol{q}$). The $\rho^{-}\rho^{-}$ terms is only non-vanishing when $\tau = -\tau'$. In addition, since $\boldsymbol{q}$ is much smaller than $|K_{+} - K_{-}|$, we can ignore the $\boldsymbol{q}$ dependence in the interaction term and replace it by the constant $V(|K_{+} - K_{-}|)$. Thus, the resulting Hamiltonian consists of two parts

$$\mathcal{H}_{\text{int}} = \mathcal{H}_0 + \mathcal{H}_J, \tag{27}$$

$\mathcal{H}_0$ contains the coupling between intravalley densities $\rho^{+}\rho^{+}$ whereas $\mathcal{H}_J$ contains the coupling between intervalley densities

$\rho^-\rho^-$. They are given explicitly by

$$\mathcal{H}_0 = \frac{V_0}{2N} \sum_{\sigma,\sigma',\tau,\tau',\boldsymbol{q}} \sum_{\mathbf{k},\mathbf{k}'\in BZ} v_{\boldsymbol{q}} \lambda^+_{\tau,\boldsymbol{q}}(\mathbf{k})[\lambda^+_{\tau',\boldsymbol{q}}(\mathbf{k}')]^* c^\dagger_{\sigma,\tau}(\mathbf{k}) c_{\sigma,\tau}(\mathbf{k}+\boldsymbol{q}) c^\dagger_{\sigma',\tau'}(\mathbf{k}'+\boldsymbol{q}) c_{\sigma',\tau'}(\mathbf{k}'), \tag{28}$$

$$\mathcal{H}_J = \frac{3J}{2N} \sum_{\sigma,\sigma',\tau,\boldsymbol{q}} \sum_{\mathbf{k},\mathbf{k}'\in BZ} \lambda^-_{\tau,\boldsymbol{q}}(\mathbf{k})[\lambda^-_{-\tau,-\boldsymbol{q}}(\mathbf{k}'+\boldsymbol{q})]^* c^\dagger_{\sigma,\tau}(\mathbf{k}) c_{\sigma,-\tau}(\mathbf{k}+\boldsymbol{q}) c^\dagger_{\sigma',-\tau}(\mathbf{k}'+\boldsymbol{q}) c_{\sigma',\tau}(\mathbf{k}'), \tag{29}$$

where the intravalley and intervalley form factors $\lambda^\pm_{\tau,\boldsymbol{q}}(\mathbf{k})$ are defined as

$$\lambda^\pm_{\tau,\boldsymbol{q}}(\mathbf{k}) = \langle u_{\tau,\mathbf{k}} | u_{\pm\tau,\mathbf{k}+\boldsymbol{q}} \rangle. \tag{30}$$

All momenta in Eq. 28 and Eq. 29 are measured in units of $q_M = \frac{4\pi\theta}{3\sqrt{3}a}$ with $v_{\boldsymbol{q}} = |q_M|/\sqrt{\boldsymbol{q}^2+\kappa^2}$ denoting the dimensionless screened Coulomb interaction with a screening length $1/\kappa$. The main source of screening is from the gate, which has the distance about 30-50 nm from the sample. The distance is comparable to the Moiré length scale, implying that the screening length can be important. In the following calculation, we would use $\kappa = 5\times 10^7$ m^{-1}. Rough estimations for V_0 and J provide the scale of the two interaction terms and are given by

$$\begin{aligned} V_0 &= \frac{e^2}{2\epsilon\epsilon_0|\Omega|q_M} = \frac{e^2\theta}{4\pi\epsilon\epsilon_0 a} \approx 176\frac{\theta^o}{\epsilon}\text{meV}, \\ J &= \frac{e^2}{2\epsilon\epsilon_0|\Omega||K-K'|} = \frac{e^2\theta^2}{4\pi\epsilon\epsilon_0 a} \approx 3.1\frac{(\theta^o)^2}{\epsilon}\text{meV}. \end{aligned} \tag{31}$$

Here, we used $|\Omega| = \frac{3\sqrt{3}a^2}{2\theta^2}$ and used θ^o to denote the value of θ in degrees. Using a value of ϵ of about 5 at twist angles around 1° yields $V_0 \approx 35$ meV and $J = 0.6$ meV. We see that the J term is significantly smaller than the V_0 term. It can be important, however, since it identifies the two separate spin-rotation symmetry for $\mathbf{K}_\pm$ valleys SU(2)$_+$ $\times$ SU(2)$_-$ down to the single spin-rotation SU(2) symmetry, while preserving valley U(1) symmetry. Thus, it can lift the degeneracy between some symmetry breaking states which are degenerate on the level of the V_0 interaction. The J term generally has the effect of favoring spin alignment and can be written in the form of inter-valley Hund's coupling as in [9].

We notice that the interaction term is invariant under the gauge transformation

$$c_{\sigma,\tau}(\mathbf{k}) \to e^{i\theta_\tau(\mathbf{k})} c_{\sigma,\tau}(\mathbf{k}), \tag{32}$$

$$\lambda_{\tau,\boldsymbol{q}}(\mathbf{k}) \to e^{i(\theta_\tau(\mathbf{k})-\theta_\tau(\mathbf{k}+\boldsymbol{q}))} \lambda_{\tau,\boldsymbol{q}}(\mathbf{k}). \tag{33}$$

Time-reversal symmetry imposes an additional constraint on the gauge transformation, $\theta_\tau(\mathbf{k}) = -\theta_\tau(-\mathbf{k})$.

Supplementary Note 3. Supplementary Note 3. Hartree-Fock calculation

Here, we provide the details for the Hartree-Fock calculation. Throughout this section, we neglect the Hund's coupling term which is discussed in the previous section and drop the superscript $\pm$ from the form factor λ such that $\lambda_{\tau,\boldsymbol{q}}(\mathbf{k}) = \lambda^{+}_{\tau,\boldsymbol{q}}(\mathbf{k})$ since we only consider the intravalley form factor here.

We now move on to the general setup for the Hartree-Fock mean field theory. Define the expectation value

$$M_{\sigma\tau,\sigma'\tau'}(\mathbf{k},\mathbf{k}') = \langle c^{\dagger}_{\sigma,\tau}(\mathbf{k}) c_{\sigma',\tau'}(\mathbf{k}')\rangle, \tag{34}$$

which we will assume to be diagonal in $\mathbf{k}$ and $\mathbf{k}'$, $M(\mathbf{k},\mathbf{k}') = \delta_{\mathbf{k},\mathbf{k}'} M(\mathbf{k})$. In the following, we will introduce the combined index $\alpha = (\sigma,\tau)$ such that $M(\mathbf{k})$ is a matrix with components $M_{\alpha,\alpha'}(\mathbf{k})$. Next, we expand the interaction (Eq. 27) in the difference $c^{\dagger}_{\alpha} c_{\alpha'} - M_{\alpha,\alpha'}$ and neglect terms beyond linear order.

The resulting mean field Hamiltonian has the form

$$\begin{aligned} \mathcal{H}_{\rm MF} &= \mathcal{H}_K + \mathcal{H}_V, \\ \mathcal{H}_K &= \sum_{\mathbf{k}} c^{\dagger}_{\mathbf{k}} [\xi(\mathbf{k}) + h_0(\mathbf{k}) + h_1(\mathbf{k})] c_{\mathbf{k}}, \\ \mathcal{H}_V &= -\frac{1}{2}\sum_{\mathbf{k}} \mathrm{tr}[h_0(\mathbf{k}) + h_1(\mathbf{k})] M^T(\mathbf{k}). \end{aligned} \tag{35}$$

Here, $c_{\mathbf{k}}$ is a column vector in the index α, $\xi(\mathbf{k})$ is a diagonal matrix containing the single particle energies $\xi_{\uparrow/\downarrow,\pm}(\mathbf{k})$ and $h_{0,1}(\mathbf{k})$ are 4×4 matrices in α given by

$$h_0 = \frac{V_0}{N} \sum_{\boldsymbol{G},\mathbf{k}'} \left\{ v_{\boldsymbol{G}} \Lambda^{+}_{\boldsymbol{G}}(\mathbf{k})\, \mathrm{tr}\, M(\mathbf{k}') [\Lambda^{+}_{\boldsymbol{G}}(\mathbf{k}')]^* - v_{\boldsymbol{G}+\mathbf{k}'} \Lambda^{+}_{\mathbf{k}'+\boldsymbol{G}}(\mathbf{k}) M^T(\mathbf{k}+\mathbf{k}') [\Lambda^{+}_{\mathbf{k}'+\boldsymbol{G}}(\mathbf{k})]^* \right\}, \tag{36}$$

and

$$h_1 = \frac{3J}{N} \sum_{\boldsymbol{G},\mathbf{k}',\tau} \left\{ P_\tau \Lambda^{-}_{\boldsymbol{G}}(\mathbf{k}) \tau_x\, \mathrm{tr}\, P_{-\tau} \Lambda^{-}_{-\boldsymbol{G}}(\mathbf{k}') \tau_x M^T(\mathbf{k}')] - P_\tau \Lambda^{-}_{\mathbf{k}'+\boldsymbol{G}}(\mathbf{k}) \tau_x M^T(\mathbf{k}+\mathbf{k}') P_{-\tau} [\Lambda^{-}_{-\mathbf{k}'-\boldsymbol{G}}(-\mathbf{k})]^T \tau_x \right\}. \tag{37}$$

The matrix $\Lambda^{\pm}_{\boldsymbol{q}}(\mathbf{k})$ simply contains the form factors defined in Eq. 30

$$[\Lambda^{\pm}_{\boldsymbol{q}}(\mathbf{k})]_{\alpha,\alpha'} = \delta_{\sigma,\sigma'} \delta_{\tau,\tau'} \lambda^{\pm}_{\tau,\boldsymbol{q}}(\mathbf{k}), \tag{38}$$

and $P_{\pm} = \frac{1}{2}(1 \pm \tau_z)$ is the projector on the $\pm$ valley with $\tau_{x,y,z}$ denoting the Pauli matrices in the valley space.

In both Eq. 36 and Eq. 37, the first term is a Hartree term whereas the second is a Fock term. Hartree terms were neglected in some of the previous mean-field studies [5, 9] since they are expected to couple only to the density which is determined by the filling in the gapped phase and is independent of the symmetry-breaking order. This is, however, not true in the presence of the form factors which are not the same for the two valleys $\lambda^{\pm}_{+,\boldsymbol{q}}(\mathbf{k}) \neq \lambda^{\pm}_{-,\boldsymbol{q}}(\mathbf{k})$. As a result, the Hartree-term also couples to the valley density and it cannot be neglected.

It is important here to point out one major difference between our approach and the one employed recently in a self-consistent Hartree-Fock mean field study in twisted bilayer graphen [10]. In that work, the Hartree-Fock corrections to the flat bands coming from all other (~ 150) bands were taken into account. Here, we will instead make the assumption that the effect of the Hartree-Fock contributions from the other bands is already included at some level in the model parameters which should be either fit to experiments or obtained from *ab initio* studies at charge neutrality [11, 12]. Thus, we only include the effects arising from filling the isolated band.

To write the self-consistency condition, we diagonalize $h_0(\mathbf{k}) + h_1(\mathbf{k})$ by introducing the variables $d_{\mathbf{k}} = U_{\mathbf{k}} c_{\mathbf{k}}$ for some unitary $U_{\mathbf{k}}$. We then impose the constraint $M_{\alpha,\alpha'}(\mathbf{k}) = \langle c^{\dagger}_{\alpha}(\mathbf{k}) c_{\alpha'}(\mathbf{k})\rangle$. In the following, we will only consider possible gapped phases at integer fillings ν. In this case, the self-consistency condition has the form

$$M(\mathbf{k}) = U^T_{\mathbf{k}} \chi U^*_{\mathbf{k}}, \tag{39}$$

where χ is a $\mathbf{k}$-independent matrix containing ν ones along the diagonal and zeroes everywhere else. This means that $M(\mathbf{k})$ is a projection operator satisfying

$$M(\mathbf{k})^2 = M(\mathbf{k}) = M(\mathbf{k})^{\dagger}, \qquad \mathrm{tr}\, M(\mathbf{k}) = \nu \tag{40}$$

$\nu = 2$	Example of $M(\mathbf{k})$	Sym. Gen.
SP	$(1+\sigma_z\tau_0)/2$	σ_z, τ_z, $\tau_x\mathcal{K}$
VP	$(1+\sigma_0\tau_z)/2$	σ_z, σ_x, $\tau_x\mathcal{K}$
SVL	$(1+\sigma_z\tau_z)/2$	σ_z, τ_z, $\sigma_x\tau_x\mathcal{K}$
IVC	$(1+\sigma_0\tau_x)/2$	σ_z, σ_x, $\tau_x\mathcal{K}$
SIVCL	$(1+\sigma_x\tau_x)/2$	σ_x, $\sigma_z\tau_z$, $\tau_x\mathcal{K}$

$\nu = 1, 3$	Example of $M(\mathbf{k})$	Sym. Gen.
SVP	$(1+\sigma_z\tau_0)(1+\sigma_0\tau_z)/4$	σ_z, τ_z
SPIVC	$(1+\sigma_z\tau_0)(1+\sigma_0\tau_x)/4$	σ_z, $\tau_x\mathcal{K}$
SVLIVC	$(1+\sigma_z\tau_z)(1+\sigma_x\tau_x)/4$	$\sigma_z\tau_z$, $\sigma_x\tau_x\mathcal{K}$

Supplementary Table I. Examples of order parameter $M(\mathbf{k})$ and corresponding independent generators of preserved symmetries for all possible translation-symmetric gapped states at half $\nu = 2$ and quarter $\nu = 1$ fillings. Note that the $M(\mathbf{k})$ can take a more general form. For example, in IVC or SIVCL, τ_x can be replaced by $c_x\tau_x + c_y\tau_y$ with $c_x^2 + c_y^2 = 1$. Also, for any spin-polarized state, σ_z can be replaced by any $\boldsymbol{\sigma} = \sin\theta\cos\phi\sigma_x + \sin\theta\sin\phi\sigma_y + \cos\theta\sigma_z$. Here, $\tau_x\mathcal{K}$ is a spinless time-reversal, where $\mathcal{K}$ is an anti-unitary symmetry. *Caveat:* For SVLIVC (which is like SVL+SIVCL) state at $\nu = 1, 3$, only a certain product structure (in this case spin S_z-locked SVL and spin S_x-locked SIVCL) would be allowed.

Our assumption that the phase is gapped has to be checked self-consistently by computing the mean field band structure

$$\epsilon_{\mathbf{k}} = \xi_{\mathbf{k}} + U_{\mathbf{k}}^\dagger h_{\mathbf{k}} U_{\mathbf{k}}, \tag{41}$$

and ensuring that correlation induced gap for filling ν defined as

$$\Delta = \min_{\mathbf{k}} \epsilon_{\nu+1,\mathbf{k}} - \max_{\mathbf{k}} \epsilon_{\nu,\mathbf{k}} \tag{42}$$

is positive. Here, we assumed that the mean field bands $\epsilon_{\alpha,\mathbf{k}}$ are sorted in order of increasing energy. ($\alpha = 1, 2, 3, 4$)

We notice that $M(\mathbf{k})$ is, in general, not gauge invariant. Instead it transforms as

$$M_{\sigma,\tau;\sigma',\tau'}(\mathbf{k}) \to e^{-i(\theta_\sigma(\mathbf{k}) - \theta_{\sigma'}(\mathbf{k}))} M_{\sigma,\tau;\sigma',\tau'}(\mathbf{k}), \tag{43}$$

under the gauge transformation (Eq. 33). In the following mean field analysis, we will choose the gauge such that $\theta_-(\mathbf{k}) = \theta_+(\mathbf{k})$ which guarantees the gauge independence of $M(\mathbf{k})$.

A. Half-filling $\nu = 2$

To understand the symmetry breaking at $\nu = 2$, we notice that the order parameter can be written as

$$M(\mathbf{k}) = \frac{1}{2}(1 + Q(\mathbf{k})), \qquad Q(\mathbf{k})^2 = 1, \quad \operatorname{tr} Q(\mathbf{k}) = 0 \tag{44}$$

$Q(\mathbf{k})$ can then be expanded in terms of the generators $\sigma_i\tau_j$ as described in the main text. In the absence of inter-valley Hund's coupling, the problem possesses an SU(2)$\times$SU(2) symmetry corresponding to independent spin rotations in each valley which are generated by $\sigma_{x,y,z}\tau_{0,z}$ in addition to U$_V$(1) valley charge conservation generated by τ_z and time-reversal symmetry given by $\mathcal{T} = \tau_x K$. Inter-valley Hund's coupling further breaks the SU(2)$\times$SU(2) to SU(2) corresponding to overall rotations. The generators can be grouped into 5 categories according to the symmetries they break as summarized in Table I. We notice that all these terms commute or anticommute with the generators of spin rotation $\sigma_{x,z}$, of U$_V$(1) valley-charge conservation τ_z and with time-reversal symmetry. In fact, when considering possible symmetry broken states in the limit of flat bands and decoupled valleys, we can always restrict ourselves to matrices $Q(\mathbf{k})$ which satisfy this requirement (for some choice of the generators of the symmetries). The reason is that such order are always energetically more favorable. To see this, consider a 'mixed' order

given by

$$Q(\mathbf{k}) = \cos\theta Q_1(\mathbf{k}) + \sin\theta Q_2(\mathbf{k}), \qquad Q_{1,2}^2 = 1, \quad \{Q_1, Q_2\} = 0 \tag{45}$$

The Fock contribution to the mean-field energy is given by

$$E_{\mathrm{HF}}[Q] = E_0 + \frac{V_0}{N}\sum_{\boldsymbol{G},\mathbf{k}'}\left\{v_{\boldsymbol{G}}\,\mathrm{tr}\,\Lambda^+_{\boldsymbol{G}}(\mathbf{k})M(\mathbf{k})\,\mathrm{tr}\,M(\mathbf{k}')[\Lambda^+_{\boldsymbol{G}}(\mathbf{k}')]^\dagger - v_{\boldsymbol{G}+\mathbf{k}'}\,\mathrm{tr}\,\Lambda^+_{\mathbf{k}'+\boldsymbol{G}}(\mathbf{k})Q(\mathbf{k}+\mathbf{k}')[\Lambda^+_{\mathbf{k}'+\boldsymbol{G}}(\mathbf{k})]^\dagger Q(\mathbf{k})\right\} \tag{46}$$

Substituting the mixed order (45), we find that the mixed term containing both Q_1 and Q_2 has to vanish since there is some symmetry generator which commutes with Q_1 and anticommutes with Q_2 (note that the form factors are invariant under all symmetries). This implies that

$$E_{\mathrm{HF}}[\cos\theta Q_1(\mathbf{k}) + \sin\theta Q_2(\mathbf{k})] = E_0 + \cos^2\theta E_{\mathrm{HF}}[Q_1] + \sin^2\theta E_{\mathrm{HF}}[Q_2] \tag{47}$$

Since the Hartree-Fock solutions has to be extrema of the Hartree-Fock energy functional, we conclude that only pure orders which either commute or anticommute with each symmetry generator are possible self-consistent solution. This justifies restricting ourselves to the list of orders provided in Table I in the main text: SP, VP, SVL, IVC, and SIVCL (such order parameters are in general **k**-dependent and may have more complicated forms than the ones written in the second column of the figure, but they have to respect the same symmetries).

If we first neglect the intervalley Hund's coupling, we notice that the mean-field energies of the SP and SVL are equal as well as the IVC and SIVCL since they are related by rotating the spin in one of the valleys. Thus, in the following discussion, we can restrict ourselves to VP, SP, and IVC orders.

1. *Valley polarized (VP) state*

A valley polarized states breaks time-reversal but preserves spin rotation and valley charge. Together with the requirement that the order parameter has the form (44), this yields

$$M_{\mathrm{VP}}(\mathbf{k}) = \frac{1}{2}\sigma_0(1+\tau_z). \tag{48}$$

The eigenvalues of $h_{\mathbf{k}}$ are given by

$$\epsilon_{\sigma,+,\mathbf{k}} = \xi_{+,\mathbf{k}} - \frac{V_0}{N}\sum_{\boldsymbol{G},\mathbf{k}'}\left\{v_{\boldsymbol{G}+\mathbf{k}'}|\lambda_{+,\mathbf{k}'+\boldsymbol{G}}(\mathbf{k})|^2 - 2v_{\boldsymbol{G}}\lambda_{+,\boldsymbol{G}}(\mathbf{k})\lambda^*_{+,\boldsymbol{G}}(\mathbf{k}')\right\}, \tag{49}$$

$$\epsilon_{\sigma,-,\mathbf{k}} = \xi_{-,\mathbf{k}} + \frac{2V_0}{N}\sum_{\boldsymbol{G},\mathbf{k}'} v_{\boldsymbol{G}}\lambda_{-,\boldsymbol{G}}(\mathbf{k})\lambda^*_{+,\boldsymbol{G}}(\mathbf{k}'). \tag{50}$$

We now need to check the correlated gap defined as

$$\Delta_{\mathrm{VP}} \equiv \min_{\mathbf{k},\sigma}\epsilon_{\sigma,-,\mathbf{k}} - \max_{\mathbf{k}',\sigma'}\epsilon_{\sigma',+,\mathbf{k}'} > 0, \tag{51}$$

is positive, so that the fully valley polarized state is a proper gapped state.

The total energy of the valley polarized state is obtained by adding the kinetic energy of the filled bands and the potential energy leading to

$$E_{\mathrm{VP}} = 2\sum_{\mathbf{k}}\xi_+(\mathbf{k}) + \frac{2V_0}{N}\sum_{\boldsymbol{G}} v_{\boldsymbol{G}}\Big|\sum_{\mathbf{k}}\lambda_{+,\boldsymbol{G}}(\mathbf{k})\Big|^2 - \frac{V_0}{N}\sum_{\boldsymbol{q},\mathbf{k}} v_{\boldsymbol{q}}|\lambda_{+,\boldsymbol{q}}(\mathbf{k})|^2. \tag{52}$$

2. Spin polarized (SP) state

Next we assume a spin polarized state along the z-direction in both valleys. Such state breaks spin-rotation but preserves time-reversal and valley charge conservation with the order parameter given by

$$M_{\mathrm{SP}}(\mathbf{k}) = \frac{1}{2}\tau_0(\sigma_0 + \sigma_z). \tag{53}$$

The energy eigenvalues are

$$\epsilon_{\uparrow,\tau,\mathbf{k}} = \xi_{\tau,\mathbf{k}} - \frac{V_0}{N}\sum_{\boldsymbol{G},\mathbf{k}'}\left\{v_{\boldsymbol{G}+\mathbf{k}'}|\lambda_{\tau,\mathbf{k}'+\boldsymbol{G}}(\mathbf{k})|^2 - v_{\boldsymbol{G}}\lambda_{\tau,\boldsymbol{G}}(\mathbf{k})\sum_{\tau'}\lambda^*_{\tau',\boldsymbol{G}}(\mathbf{k}')\right\}, \tag{54}$$

$$\epsilon_{\downarrow,\tau,\mathbf{k}} = \xi_{\tau,\mathbf{k}} + \frac{V_0}{N}\sum_{\boldsymbol{G}} v_{\boldsymbol{G}}\lambda_{\tau,\boldsymbol{G}}(\mathbf{k})\sum_{\tau',\mathbf{k}'}\lambda^*_{\tau',\boldsymbol{G}}(\mathbf{k}'). \tag{55}$$

We also require the gap $\Delta_{\mathrm{SP}} = \min_{\mathbf{k},\tau}\epsilon_{\uparrow,\tau,\mathbf{k}} - \max_{\mathbf{k}',\tau'}\epsilon_{\uparrow,\tau',\mathbf{k}'}$ to be positive so that the spin polarized state is a proper gapped state.

The total energy of the spin polarized state is given by

$$E_{\mathrm{SP}} = \sum_{\tau,\mathbf{k}}\xi_\tau(\mathbf{k}) + \frac{V_0}{2N}\sum_{\boldsymbol{G}} v_{\boldsymbol{G}}\Big|\sum_{\tau,\mathbf{k}}\lambda_{\tau,\boldsymbol{G}}(\mathbf{k})\Big|^2 - \frac{V_0}{2N}\sum_{\boldsymbol{q},\tau,\mathbf{k}} v_{\boldsymbol{q}}|\lambda_{\tau,\boldsymbol{q}}(\mathbf{k})|^2. \tag{56}$$

Comparing to the VP state, we find that the two phases have exactly the same ground state energy. This follows from time-reversal symmetry which implies that $\xi_-(\mathbf{k}) = \xi_+(-\mathbf{k})$ and $\lambda_{-,\boldsymbol{G}}(\mathbf{k}) = \lambda_{+,\boldsymbol{G}}(-\mathbf{k})$ as well as $|\lambda_{-,\boldsymbol{q}}(\mathbf{k})| = |\lambda_{+,-\boldsymbol{q}}(-\mathbf{k})|$. Using the relation $\sum_{\mathbf{k}}\lambda_{+,\boldsymbol{G}}(\mathbf{k}) = \sum_{\mathbf{k}}\lambda_{-,\boldsymbol{G}}(\mathbf{k})$, one can show that the total energies as well as the gaps are the same for VP and SP states.

3. Intervalley coherent (IVC) order

The intervalley coherent order parameter is given by

$$M_{\mathrm{IVC}}(\mathbf{k}) = \sigma_0\begin{pmatrix}\cos^2\frac{\theta_{\mathbf{k}}}{2} & \frac{1}{2}\sin\theta_{\mathbf{k}}e^{-i\phi_{\mathbf{k}}}\\ \frac{1}{2}\sin\theta_{\mathbf{k}}e^{i\phi_{\mathbf{k}}} & \sin^2\frac{\theta_{\mathbf{k}}}{2}\end{pmatrix}. \tag{57}$$

We note that it is not possible in general to take the fully polarized limit in the $x-y$ plane and at the same time fulfill the self-consistency conditions. Hence, we include a small z valley polarization parametrized by the angle $\theta_{\mathbf{k}}$. We notice that the state (Eq. 57) will not break time-reversal symmetry provided that $\theta_{-\mathbf{k}} = \pi - \theta_{\mathbf{k}}$ and $\phi_{-\mathbf{k}} = -\phi_{\mathbf{k}}$ which implies that the average valley polarization $\sum_{\mathbf{k}}\cos\theta_{\mathbf{k}}$ vanishes.

The mean field Hamiltonian has the form

$$h_{\mathbf{k}} = \begin{pmatrix} f_{\mathbf{k}} + A_{\mathbf{k}} & B_{\mathbf{k}}\\ B^*_{\mathbf{k}} & f_{\mathbf{k}} - A_{\mathbf{k}}\end{pmatrix}, \tag{58}$$

with $f_{\mathbf{k}}$, $A_{\mathbf{k}}$, $B_{\mathbf{k}}$ given by

$$f_{\mathbf{k}} = \sum_\tau\left\{\frac{1}{2}\xi_\tau(\mathbf{k}) - \frac{V_0}{4N}\sum_{\boldsymbol{q}} v_{\boldsymbol{q}}|\lambda_{\tau,\boldsymbol{q}}(\mathbf{k})|^2(1+\cos\theta_{\mathbf{k}+\boldsymbol{q}}) + \frac{V_0}{2N}\sum_{\boldsymbol{G},\mathbf{k}',\tau'} v_{\boldsymbol{G}}\lambda_{\tau,\boldsymbol{G}}(\mathbf{k})\lambda_{\tau',\boldsymbol{G}}(\mathbf{k}')(1+\tau'\cos\theta_{\mathbf{k}'})\right\}, \tag{59}$$

$$A_{\mathbf{k}} = \sum_\tau \tau\left\{\frac{1}{2}\xi_\tau(\mathbf{k}) - \frac{V_0}{4N}\sum_{\boldsymbol{q}} v_{\boldsymbol{q}}|\lambda_{\tau,\boldsymbol{q}}(\mathbf{k})|^2(1+\tau\cos\theta_{\mathbf{k}+\boldsymbol{q}}) + \frac{V_0}{2N}\sum_{\boldsymbol{G},\mathbf{k}',\tau'} v_{\boldsymbol{G}}\lambda_{\tau,\boldsymbol{G}}(\mathbf{k})\lambda_{\tau',\boldsymbol{G}}(\mathbf{k}')(1+\tau'\cos\theta_{\mathbf{k}'})\right\}, \tag{60}$$

$$B_{\mathbf{k}} = -\frac{V_0}{2N}\sum_{\boldsymbol{q}} v_{\boldsymbol{q}}\lambda_{+,\boldsymbol{q}}(\mathbf{k})\lambda^*_{-,\boldsymbol{q}}(\mathbf{k})\sin\theta_{\mathbf{k}+\mathbf{k}'}e^{-i\phi_{\mathbf{k}+\mathbf{k}'}}. \tag{61}$$

The self-consistency condition reads

$$\tan\phi_{\mathbf{k}} = -\frac{\operatorname{Im} B_{\mathbf{k}}}{\operatorname{Re} B_{\mathbf{k}}}, \qquad \tan\theta_{\mathbf{k}} = -\frac{|B_{\mathbf{k}}|}{A_{\mathbf{k}}}, \tag{62}$$

where energy eigenvalues are given by

$$\epsilon_{\pm}(\mathbf{k}) = f_{\mathbf{k}} \pm \sqrt{A_{\mathbf{k}}^2 + |B_{\mathbf{k}}|^2}, \tag{63}$$

with the gap given by $\Delta_{\rm IVC} = \min_{\mathbf{k}} \epsilon_{+,\mathbf{k}} - \max_{\mathbf{k}'} \epsilon_{-,\mathbf{k}'}$ which should be positive for a proper gapped phase. The results of the energy competition between the VP/SP phase and the IVC state obtained by numerically solving the self-consistency equation are given in the main text.

4. Effect of intervalley Hund's coupling

As we have seen above, the three distinct states with spin polarization, valley polarization or spin-valley locking are degenerate in the absence of intervalley Hund's and their energy is always lower than the energy of the valley off-diagonal orders (IVC and SIVCL). In the following, we want to investigate the effect of intervalley Hund's coupling on these three states. Since this term J is much smaller than the main part of the interaction V_0, it suffices to compute it for the three valley-diagonal orders since the valley off-diagonal orders are already energetically unfavorable on the level of V_0. Substituting in (37) we find that

$$E_J = \begin{cases} -\frac{3J}{N}\sum_{\tau,\mathbf{k},\boldsymbol{q}} |\lambda^-_{\tau,\boldsymbol{q}}(\mathbf{k})|^2 & : \text{SP} \\ 0 & : \text{VP} \\ \frac{3J}{N}\sum_{\tau,\mathbf{k},\boldsymbol{q}} |\lambda^-_{\tau,\boldsymbol{q}}(\mathbf{k})|^2 & : \text{SVL} \end{cases} \tag{64}$$

B. Quarter-filling $\nu = 1$

At quarter filling, $\nu = 1$ (similarly for $\nu = 3$, with some caveats), we can always write the order parameter as

$$M(\mathbf{k}) = \frac{1}{4}(1 + Q_1(\mathbf{k}))(1 + Q_2(\mathbf{k})), \qquad Q_{1,2}(\mathbf{k})^2 = 1, \quad \operatorname{tr} Q_{1,2}(\mathbf{k}) = 0, \quad [Q_1(\mathbf{k}), Q_2(\mathbf{k})] = 0 \tag{65}$$

This leads to three distinct possibilities: (i) $Q_1 = \sigma_{x,y,z}\tau_0$ and $Q_2 = \sigma_0\tau_z$ which corresponds to a spin and valley polarized state, (ii) $Q_1 = \sigma_{x,y,z}\tau_0$ and $Q_2 = \sigma_0\tau_{x,y}$ which corresponds to a spin-polarized IVC, and (iii) $Q_1 = \sigma_z\tau_z$ and $Q_2 = \sigma_x\tau_x, \sigma_y\tau_y$ which correspond to a spin-valley locked IVC.

The SPIVC and SVLIVC are related by a spin rotation in one of the valleys, thus we can focus only on the competition between SVP and SPIVC. Compared to the VP vs IVC states at half-filling these differ by a factor of 2 in the Fock energy and a factor of 4 in the Hartree energy. Since the former is the main deciding factor in the competition between the phases, the results for the gaps and energy difference between SVP and SPIVC at quarter filling are very similar to those between VP and IVC at half-filling.

Supplementary Note 4. PERTURBATIVE SOLUTION AND COMPETITION BETWEEN VP/SP AND IVC

In this section, we would like to discuss the competition between inter-valley coherent order and valley/spin polarized order in a more general setting that is not too sensitive to the details of the model parameters. To this end, it is useful to derive an approximate solution to the self-consistency equations and compute an analytic expression for the energy difference between the IVC phase and the VP/SP phase.

In order to make progress analytically, we can write the IVC order parameter as

$$\theta_{\mathbf{k}} = \frac{\pi}{2} + \gamma_{\mathbf{k}}, \qquad \phi_{\mathbf{k}} = \beta_{\mathbf{k}}. \tag{66}$$

where $\gamma_{\mathbf{k}} \sim \beta_{\mathbf{k}} \sim \delta \ll 1$. This approximation can be justified as follows: the starting symmetry of the isolated band is SU(4) which is broken to SU(2) $\times$ SU(2) due to the asymmetry between the two valley in energies and form factors ($\xi_+(\mathbf{k}) \neq \xi_-(\mathbf{k})$, $\lambda_{+,\boldsymbol{q}}(\mathbf{k}) \neq \lambda_{-,\boldsymbol{q}}(\mathbf{k})$). In the following, we will assume that breaking SU(4) to SU(2) $\times$ SU(2) is not very strong so that the deviation from the situation where the valleys are identical is weak. This condition can be written more explicitly as the requirement that $\frac{|\xi_+(\mathbf{k})-\xi_-(\mathbf{k})|}{V_0} \sim |\lambda_{+,\boldsymbol{q}}(\mathbf{k}) - \lambda_{-,\boldsymbol{q}}(\mathbf{k})| \sim \delta \ll 1$. The first part is guaranteed by the small bandwidth whereas the second one can be checked numerically and shown to hold at least for most values of $\mathbf{k}$ and $\boldsymbol{q}$. This is equivalent to expanding in time-reversal symmetry breaking terms within each valley.

The variables $\gamma_{\mathbf{k}}$ and $\beta_{\mathbf{k}}$ can be obtained by solving a linearized version of the self-consistency equation as follows. We start by expanding $\theta_{\mathbf{k}}$ and $\phi_{\mathbf{k}}$ in terms of small deviations δ from a perfect IVC state in the τ_x as shown in Eq. 66. Substituting in Eq. 62 and expanding to leading order in δ yields the following set of linear equations given by

$$\gamma_{\mathbf{k}} b_{\mathbf{k}} - \sum_{\mathbf{k}'} F_{\mathbf{k},\mathbf{k}'} \gamma_{\mathbf{k}'} = a_{\mathbf{k}}, \qquad \beta_{\mathbf{k}} b_{\mathbf{k}} - \sum_{\mathbf{k}'} F_{\mathbf{k},\mathbf{k}'} \beta_{\mathbf{k}'} = -\operatorname{Im} b_{\mathbf{k}}, \tag{67}$$

where $a_{\mathbf{k}}$ is given by

$$a_{\mathbf{k}} = \sum_{\tau} \tau \left\{ \frac{\xi_\tau(\mathbf{k})}{U} - \frac{1}{2N} \sum_{\boldsymbol{q}} v_{\boldsymbol{q}} |\lambda_{\tau,\boldsymbol{q}}(\mathbf{k})|^2 + \frac{1}{N} \sum_{\boldsymbol{G},\mathbf{k}',\tau'} v_{\boldsymbol{G}} \lambda_{\tau,\boldsymbol{G}}(\mathbf{k}) \lambda_{\tau',\boldsymbol{G}}(\mathbf{k}') \right\}, \tag{68}$$

and $F_{\mathbf{k},\mathbf{k}'}$ and $b_{\mathbf{k}}$ are given by

$$F_{\mathbf{k},\mathbf{k}'} = \frac{1}{N} \sum_{\boldsymbol{G}} v_{\boldsymbol{G}+\mathbf{k}'-\mathbf{k}} |\lambda_{+,\boldsymbol{G}+\mathbf{k}'-\mathbf{k}}(\mathbf{k})|^2, \qquad b_{\mathbf{k}} = \frac{1}{N} \sum_{\boldsymbol{q}} v_{\boldsymbol{q}} \lambda_{+,\boldsymbol{q}}(\mathbf{k}) \lambda^*_{-,\boldsymbol{q}}(\mathbf{k}). \tag{69}$$

We notice that $a_{\mathbf{k}}$ and $\operatorname{Im} b_{\mathbf{k}}$ are of order δ. Substituting in the expression for the energy, the energy difference between the IVC state and the SP/VP state can be written (up to second order in δ) as

$$\frac{E_{\mathrm{IVC}} - E_{\mathrm{SP}}}{V_0} = \frac{1}{4N} \sum_{\mathbf{k},\boldsymbol{q}} v_{\boldsymbol{q}} \Big| \lambda_{+,\boldsymbol{q}}(\mathbf{k}) - \lambda_{-,\boldsymbol{q}}(\mathbf{k}) \Big|^2 + \frac{1}{2} \sum_{\mathbf{k},\mathbf{k}'} \beta_{\mathbf{k}} F_{\mathbf{k},\mathbf{k}'} \beta_{\mathbf{k}'} - \frac{1}{2} \sum_{\mathbf{k}} b_{\mathbf{k}} (\gamma_{\mathbf{k}}^2 + \beta_{\mathbf{k}}^2), \tag{70}$$

where $F_{\mathbf{k},\mathbf{k}'}$ and $b_{\mathbf{k}}$ are defined in (69). The first term in Eq. 70 reproduces the non self-consistent Hartree-Fock energies obtained in Ref. [9] in which case VP/SP is always favored to IVC.

The second and third terms are corrections coming from solving the self-consistency condition. It is instructive to reproduce the results of Ref. [5] which considers the simplified setting where all form factors are taken equal to 1. In addition, $v_{\boldsymbol{q}}$ was taken equal to a constant which is cutoff at large momenta $\boldsymbol{q} \sim \Lambda$ yielding the interaction strength $g = \frac{V_0}{2N} \sum_{|\boldsymbol{q}|<\Lambda} = \frac{V_0}{2} \sum_{|\boldsymbol{G}|<\Lambda}$. In this case, $\gamma_{\mathbf{k}} = \frac{\xi_+(\mathbf{k}) - \xi_-(\mathbf{k})}{2g}$ and $\beta_{\mathbf{k}} = 0$ leading to

$$E_{\mathrm{IVC}} - E_{\mathrm{SP}} = -\frac{1}{4g} \sum_{\mathbf{k}} [\xi_+(\mathbf{k}) - \xi_-(\mathbf{k})]^2, \tag{71}$$

which implies that the IVC phase is energetically favored to the VP/SP phase in agreement with the conclusion of Ref. [5] [2].

Our result (Eq. 70) interpolates between these two limits with the first two terms favoring spin or valley polarization and the last term favoring intervalley coherence. The competition between SP/VP and IVC is then settled by the details of the band

[2] The result differs by a factor of 2 due to the incorrect way the large g limit was implemented [5].

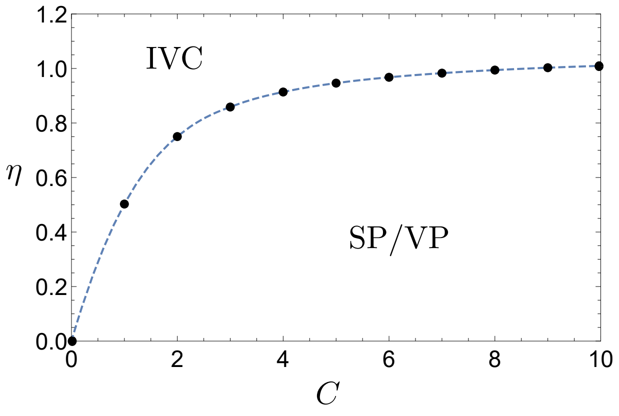

Supplementary Figure 2. Illustration of the phase diagram obtained from the perturbative solution to the self-consistency equation with the simplified form factor (Eq. 72) as a function of Chern number and $\eta = (t/V_0)\sin\phi$ which corresponds to the product of the bandwidth (relative to the Coulomb scale) and time-reversal symmetry breaking within each valley. We can see that any non-zero Chern number favors valley or spin polarization over intervalley coherent order as long as the bandwidth is not very large.

structure, form factors and interaction. We notice that this expression underestimates the energy of the IVC states when the bands have non-zero Chern number. In this case, it was shown in Ref. [13] that vortices in the IVC order parameter are unavoidable. The existence of vortices is neglected in the expansion (Eq. 66) which assumes that $\phi_{\mathbf{k}}$ is small everywhere. This implies that the expression (Eq. 70) underestimates the IVC ground state energy for non-zero Chern number.

In order to gain some insights about what parameters control this competition, let us consider a very simplified setting where the Berry curvature is uniform in momentum space with the form factor assuming the simple form [9]

$$\lambda_{\pm,\boldsymbol{q}}(\mathbf{k}) = e^{-\frac{\alpha}{4}\boldsymbol{q}^2 \pm i\frac{B}{2}\mathbf{k}\wedge\boldsymbol{q}}, \qquad B = \frac{2\pi C}{|A_{\rm BZ}|}, \tag{72}$$

Here, C is the Chern number for the $+$ valley and the parameter α determines how quickly the form factor decays with $\boldsymbol{q}$ which we take equal to $2\pi/A_{\rm BZ}$ to reproduce the Landau level form factors for $C = 1$. These form factors would be obtained in a Landau level if it is folded into a Brillouin zone of the lattice with the flux density one [14]. In addition, we will consider a very simple form of the dispersion corresponding to nearest neighbour tight-binding model on a triangular lattice with hopping amplitude $te^{\pm i\phi}$ for the $\pm$ valleys. For $C = 0$, we know that it is possible to write such a tight-binding model. For non-zero C, it is generally impossible to write such tight binding model. However, we can still use the same resulting dispersion and assume that the non-zero Chern number only affects the form factors. This will enable us to disentangle the effects of the band dispersion from those related to band topology.

For the form factors given in Eq. 72, the self-consistency equations can be solved by performing Fourier transform to real space. Following a series of straightforward steps, we get

$$\frac{E_{\rm IVC} - E_{\rm SP}}{V_0 N} \propto \left\{ \frac{1}{N}\sum_{\mathbf{k}} \left[1 - e^{-\frac{B^2\mathbf{k}^2}{2\alpha}} I_0\left(\frac{B^2\mathbf{k}^2}{2\alpha}\right)\right] - \frac{3\alpha A_{\rm BZ}^2 \eta^2}{2\pi^3\left(1 - e^{-\frac{8\pi^2}{9\alpha}} I_0\left(\frac{8\pi^2}{9\alpha}\right)\right)^2} \right\} \tag{73}$$

where $I_b(x)$ is the modified Bessel function of the first kind and $\eta = (t/V_0)\sin\phi$. The proportionality here indicates that we have dropped a constant positive factor given by $\frac{\pi}{|A_{\rm BZ}|}\sqrt{\frac{\pi}{\alpha}}$ which does not influence the competition between the two phases.

The expression (Eq. 73) depends only on two dimensionless parameters: (i) the Chern number C and (ii) η which measures the bandwidth relative to the interaction strength multiplied by the strength of time-reversal symmetry breaking within each valley. The first term in Eq. 73 is always positive and favors SP/VP state. It vanishes for zero Chern number and increases as the Chern number increases. This suggests that increasing the Chern number favors valley/spin polarization over intervalley coherent order. The second term, on the other hand, favors IVC and increases with increasing the bandwidth or the time-reversal symmetry breaking within each valley.

The phase diagram for different values of C and η is given in Fig. 2. For $C = 0$, IVC order always wins. This is an artifact of our simple choice for the form factors which corresponds to uniform Berry curvature. In a more realistic model where the Berry curvature vanishes on average but does not vanish everywhere, we expect some region of VP/SP. This is expected to be particularly manifest in the vicinity of topological phase transitions where the valley Chern number changes leading to a large concentration of the Berry curvature at some momenta. For $C \neq 0$, we find that VP/SP is always favored for relatively small

values of the bandwidth whereas IVC is favored for relatively large values. Since our approach underestimates the IVC energy for non-zero Chern number (since it ignores vortices [13]), we expect the transition from VP/SP to IVC to happen at even larger values of η implying that VP/SP is the most energetically favorable insulator at half-filling whenever the bandwidth is relatively narrow.

Supplementary Note 5. SPIN-TRIPLET SUPERCONDUCTIVITY

In the following, we provide some details on the discussion related to spin-triplet superconductivity in the main text. The interaction term

$$\mathcal{H} = \sum_{\mathbf{k},\tau,\sigma} c^{\dagger}_{\sigma,\tau,\mathbf{k}} \xi_{\sigma,\tau,\mathbf{k}} c_{\sigma,\tau,\mathbf{k}} - g \sum_{\boldsymbol{q}} \boldsymbol{S_q} \cdot \boldsymbol{S_{-q}} \tag{74}$$

can be rewritten as

$$g_{\alpha,\beta;\gamma,\delta} \sum_{\mathbf{k},\mathbf{k}',\tau,\tau'} c^{\dagger}_{\alpha,\tau,\mathbf{k}+\boldsymbol{q}} c^{\dagger}_{\gamma,\tau',\mathbf{k}'-\boldsymbol{q}} c_{\beta,\tau,\mathbf{k}} c_{\delta,\tau',\mathbf{k}'} \tag{75}$$

with $g_{\alpha,\beta;\gamma,\delta} = \sum_a \sigma^a_{\alpha\beta}\sigma^a_{\gamma\delta}$. When performing the BCS decoupling, we restrict ourselves to pairing between time-reversed pairing which corresponds to $\mathbf{k}' = -\mathbf{k}$ and $\tau' = -\tau$. In this case, we can define the gap function $\Delta_{\alpha,\beta,\tau,\tau'\mathbf{k}} = \delta_{\tau,-\tau'} \langle c^{\dagger}_{\alpha,\tau,\mathbf{k}} c^{\dagger}_{\beta,\tau',-\mathbf{k}} \rangle$, which satisfies the linearized BCS equation

$$\sum_{\mathbf{k}'\in\mathrm{FS}} v_{\mathrm{F}}(\mathbf{k}')^{-1} g_{\alpha,\beta;\gamma,\delta} \Delta_{\beta,\delta,\tau,\tau',\mathbf{k}'} = \lambda \Delta_{\alpha,\gamma,\tau,\tau',\mathbf{k}} \tag{76}$$

where $v_{\mathrm{F}}(\mathbf{k})$ is the Fermi velocity at point $\mathbf{k}$ on the Fermi s surface $v_{\mathrm{F}}(\mathbf{k}) = |\nabla_{\mathbf{k}}\epsilon_{\mathbf{k}}|$. Choosing $\Delta_{\mathbf{k}}$ to be $\mathbf{k}$-independent, we can simplify (Eq. 76)

$$\boldsymbol{\sigma} \cdot (\Delta \boldsymbol{\sigma}^T) = \tilde{\lambda}\Delta \tag{77}$$

where $\tilde{\lambda}$ is related to λ by some constant rescaling (coming from the Fermi surface integral), $\boldsymbol{\sigma}$ is the Pauli matrix vector in spin space and Δ is a matrix in spin and valley spaces. As discussed in the main text, intervalley pairing is proportional to τ_x or τ_y which corresponds to valley triplet or singlet respectively, which, due to the overall antisymmetry of the gap function, implies the former scenario corresponds to a spin-singlet $i\sigma_y$ whereas the latter corresponds to a spin-triplet $i\sigma_y \boldsymbol{d}\cdot\boldsymbol{\sigma}$. Here, $\boldsymbol{d}$ is the vector which captures the direction of the spin state.

The symmetry of the superconducting order parameter is obtained by finding the pairing channel for which $\tilde{\lambda}$ is positive and maximum. Substituting the spin-singlet and triplet gap functions in (Eq. 77) yields

$$\boldsymbol{\sigma} \cdot (i\sigma_y \tau_x \boldsymbol{\sigma}^T) = -3 i\sigma_y \tau_x \to \tilde{\lambda}_s = -3 \tag{78}$$

$$\boldsymbol{\sigma} \cdot (i\sigma_y \boldsymbol{d}\cdot\boldsymbol{\sigma} \tau_y \boldsymbol{\sigma}^T) = i\sigma_y \boldsymbol{d}\cdot\boldsymbol{\sigma}\tau_y \to \tilde{\lambda}_t = +1 \tag{79}$$

which implies a valley-singlet spin-triplet superconductor.

Supplementary Note 6. DEPENDENCE OF T_c ON MAGNETIC FIELD

In the following, we will write a simple mean field theory to relate the parameters in the Ginzburg-Landau free energy in Eq. 6 of the main text to the microscopic parameters. We start by writing the following imaginary time mean-field action

$$S = \int_0^\beta d\tau \sum_{\mathbf{k}} \left[\psi_{\mathbf{k}}^\dagger (\partial_\tau + \xi_{\mathbf{k}} + \mu_B \mathbf{B} \cdot [-\chi \boldsymbol{\sigma} + \boldsymbol{g}_{\mathbf{k}}]) \psi_{\mathbf{k}} + \frac{1}{2} \psi_{-\mathbf{k}}^T \Delta_{\mathbf{k}} \psi_{\mathbf{k}} + \frac{1}{2} \psi_{\mathbf{k}}^\dagger \Delta_{\mathbf{k}}^\dagger \psi_{-\mathbf{k}}^* \right] + \frac{\beta}{2g} \sum_{\mathbf{k}} \operatorname{tr} \Delta_{\mathbf{k}} \Delta_{\mathbf{k}}^\dagger. \tag{80}$$

Here, ψ is a (grassman-valued) spinor in valley and spin spaces, σ and τ are Pauli matrices for the spin and valley degrees of freedom, respectively. χ is dimensionless magnetic susceptibility and Δ is a matrix in the valley and spin spaces. Following the discussion of the main text, we take $\Delta_{\mathbf{k}}$ to be $\mathbf{k}$-independent, spin-triplet and valley singlet

$$\Delta_{\mathbf{k}} = i\sigma_y \boldsymbol{d} \cdot \boldsymbol{\sigma} \tau_y. \tag{81}$$

The magnetic field enters (Eq. 80) through Zeeman and orbital couplings with the $\mathbf{k}$-dependent g-factor arising form the orbital effect (see the main text). (In (Eq. 80), $\boldsymbol{g}_{\mathbf{k}}$ is a diagonal matrix in spin and valley spaces given by $\sigma_0 \mathrm{diag}(\boldsymbol{g}_{+,\mathbf{k}}, \boldsymbol{g}_{-,\mathbf{k}})_\tau$). If the parent state is either a weak ferromagnet or close to a ferromagnetic quantum critical point which we anticipate to be the case, then dimensional susceptbtility χ can be relatively large and cannot be put to 1.

We can go now to matsubara frequency by writing

$$\psi(\tau) = \frac{1}{\sqrt{\beta}} \sum_{\omega_n} e^{i\omega_n \tau} \psi_n, \qquad \omega_n = (2n+1)\pi/\beta, \tag{82}$$

leading to

$$S = \frac{2\beta}{g} \sum_{\mathbf{k}} \boldsymbol{d}_{\mathbf{k}} \cdot \boldsymbol{d}_{\mathbf{k}}^* + \frac{1}{2} \sum_{p=(\omega_n,\mathbf{k})} \begin{pmatrix} \psi_p^\dagger & \psi_{-p}^T \end{pmatrix} \begin{pmatrix} G_p^{-1} - \mu_B \chi \boldsymbol{\sigma} \cdot \mathbf{B} + \mu_B \boldsymbol{g}_{\mathbf{k}} \cdot \mathbf{B} & \Delta_{\mathbf{k}}^\dagger \\ \Delta_{\mathbf{k}} & -G_{-p}^{-1} + \mu_B \chi \boldsymbol{\sigma}^T \cdot \mathbf{B} - \mu_B \boldsymbol{g}_{-\mathbf{k}} \cdot \mathbf{B} \end{pmatrix} \begin{pmatrix} \psi_p \\ \psi_{-p}^* \end{pmatrix}. \tag{83}$$

Here, we introduced the Green's function G_p as

$$G_p = \frac{1}{i\omega_n + \xi_{\mathbf{k}}}. \tag{84}$$

where $\xi_{\mathbf{k}}$ depends on the valley index such that $\xi_{+,-\mathbf{k}} = \xi_{-,\mathbf{k}}$. The fermions can be integrated out leading to a Pfafian which can be written in the exponential as the logarithm of the trace of some operator. The resulting free energy can be expanded in powers of $\mathbf{B}$ and Δ.

The term proportional to $\Delta\Delta^\dagger$ provides the standard BCS instability which is given by

$$F_{\Delta\Delta^\dagger} = -\frac{1}{2\beta} \sum_{p=(\omega_n,\mathbf{k})} \operatorname{tr} \Delta_{\mathbf{k}} G_p \Delta_{\mathbf{k}}^\dagger G_{-p} = -\frac{2\boldsymbol{d} \cdot \boldsymbol{d}^*}{\beta} \int d\xi N(\xi) \sum_{\omega_n} \frac{1}{\omega_n^2 + \xi^2} = -\frac{2\boldsymbol{d} \cdot \boldsymbol{d}^*}{\beta} \int d\xi N(\xi) f(\xi), \tag{85}$$

where $f(\xi)$ is defined as

$$f(\xi) = \sum_{\omega_n} \frac{1}{\omega_n^2 + \xi^2}. \tag{86}$$

The integral over ξ is cut off by the bandwidth Λ. However, we can choose to perform the ξ integral before the frequency sum in which case, the integral is automatically cut off by ω_n so that it can be extended to infinity with the cutoff Λ moved to the ω_n sum instead. This leads to

$$\int d\xi N(\xi) f(\xi) = \sum_{|\omega_n| < \Lambda} \frac{\pi N(0)}{|\omega_n|} \approx \beta N(0) \int_{1/\beta}^{\Lambda} d\omega \frac{1}{\omega} = \beta N(0) \log \beta\Lambda. \tag{87}$$

The final result is given by

$$F_{\Delta\Delta^\dagger} = -2N(0) \log \beta\Lambda \, \boldsymbol{d} \cdot \boldsymbol{d}^*. \tag{88}$$

The term proportional to $\Delta\Delta^\dagger \mathbf{B}\cdot\boldsymbol{\sigma}$ is given by

$$\begin{aligned} F_{\Delta\Delta^\dagger\mathbf{B}\cdot\boldsymbol{\sigma}} &= -\frac{\mu_B\chi}{2\beta}\sum_{p=(\omega_n,\mathbf{k})}\mathrm{tr}\left(\Delta G_p\Delta^\dagger G_{-p}\mathbf{B}\cdot\boldsymbol{\sigma}^T G_{-p} + \Delta G_p\mathbf{B}\cdot\boldsymbol{\sigma}G_p\Delta^\dagger G_{-p}\right) \\ &= \frac{4i\mu_B\chi}{\beta}\mathbf{B}\cdot(\boldsymbol{d}^*\times\boldsymbol{d})\int d\xi N(\xi)f'(\xi) = -4i\mu_B\chi N'(0)\log\beta\Lambda\,\mathbf{B}\cdot(\boldsymbol{d}^*\times\boldsymbol{d}). \end{aligned} \tag{89}$$

The linear term corresponding to the orbital effect $\Delta\Delta^\dagger\mathbf{B}\cdot\boldsymbol{g}$ vanishes due to time-reversal symmetry which can be seen as follows

$$\begin{aligned} F_{\Delta\Delta^\dagger\mathbf{B}\cdot\boldsymbol{g}} &= -\frac{\mu_B}{2\beta}\sum_{p=(\omega_n,\mathbf{k})}\mathrm{tr}\left(\Delta G_p\Delta^\dagger G_{-p}\tau_z\mathbf{B}\cdot\boldsymbol{g}_{-\mathbf{k}}G_{-p} + \Delta G_p\tau_z\mathbf{B}\cdot\boldsymbol{g}_{\mathbf{k}}G_p\Delta^\dagger G_{-p}\right) \\ &= 4\frac{\mu_B\boldsymbol{d}\cdot\boldsymbol{d}^*}{\beta}\sum_{\tau_{1,2}=\pm}\int_{\mathrm{FS}} d\mathbf{k}(\mathbf{B}\cdot\boldsymbol{g}_{\tau_1,\tau_2\mathbf{k}})\int d\xi N(\xi)f'(\xi) = 0. \end{aligned} \tag{90}$$

The last equality follows from the fact that $\boldsymbol{g}_{\tau,\mathbf{k}}$ is odd under time-reversal symmetry $\boldsymbol{g}_{+,-\mathbf{k}} = -\boldsymbol{g}_{-,\mathbf{k}}$.

The term proportional to $\Delta\Delta^\dagger(\mathbf{B}\cdot\boldsymbol{\sigma})^2$ is given by

$$\begin{aligned} F_{\Delta\Delta^\dagger(\mathbf{B}\cdot\boldsymbol{\sigma})^2} &= -\frac{\mu_B^2\chi^2}{2\beta}\sum_{p=(\omega_n,\mathbf{k})}\mathrm{tr}\left(\Delta G_p\Delta^\dagger G_{-p}\mathbf{B}\cdot\boldsymbol{\sigma}^T G_{-p}\mathbf{B}\cdot\boldsymbol{\sigma}^T G_{-p} + \Delta G_p\mathbf{B}\cdot\boldsymbol{\sigma}G_p\Delta^\dagger G_{-p}\mathbf{B}\cdot\boldsymbol{\sigma}^T G_{-p}\right. \\ &\qquad \left. +\Delta G_p\mathbf{B}\cdot\boldsymbol{\sigma}G_p\mathbf{B}\cdot\boldsymbol{\sigma}G_p\Delta^\dagger G_{-p}\right) \\ &= -2\frac{\mu_B^2\chi^2}{\beta}\mathbf{B}^2\boldsymbol{d}\cdot\boldsymbol{d}^*\sum_{p=(\omega_n,\mathbf{k})}(G_pG_{-p}^3 + G_p^2G_{-p}^2 + G_p^3G_{-p}) + 4\frac{\mu_B^2\chi^2}{\beta}(\boldsymbol{d}\cdot\mathbf{B})(\boldsymbol{d}^*\cdot\mathbf{B})\sum_{p=(\omega_n,\mathbf{k})}G_p^2G_{-p}^2. \end{aligned} \tag{91}$$

The first term can be simplified by noting that

$$\sum_{p=(\omega_n,\mathbf{k})}(G_pG_{-p}^3 + G_p^2G_{-p}^2 + G_p^3G_{-p}) = \frac{1}{2}\int d\xi N(\xi)f''(\xi) = \frac{1}{2}N''(0)\beta\log\Lambda\beta, \tag{92}$$

whereas the second term can be evaluated as

$$\sum_{|\omega_n|<\Lambda}\int d\xi\frac{N(\xi)}{(\omega_n^2+\xi^2)^2} = \frac{\beta}{2}N(0)\int_{1/\beta}^{\Lambda}d\omega\frac{1}{\omega^3} \approx \frac{\beta^3}{4}N(0), \tag{93}$$

leading to

$$F_{\Delta\Delta^\dagger(\mathbf{B}\cdot\boldsymbol{\sigma})^2} = -\mu_B^2\chi^2\mathbf{B}^2N''(0)\log\Lambda\beta(\boldsymbol{d}\cdot\boldsymbol{d}^*) + \mu_B^2\chi^2\beta^2N(0)(\boldsymbol{d}\cdot\mathbf{B})(\boldsymbol{d}^*\cdot\mathbf{B}). \tag{94}$$

The term proportional to $\Delta\Delta^\dagger(\mathbf{B}\cdot\boldsymbol{g})^2$ is given by

$$\begin{aligned} F_{\Delta\Delta^\dagger(\mathbf{B}\cdot\boldsymbol{g})^2} &= -\frac{\mu_B^2}{2\beta}\sum_{p=(\omega_n,\mathbf{k})}\mathrm{tr}\left(\Delta G_p\Delta^\dagger G_{-p}\mathbf{B}\cdot\boldsymbol{g}_{-\mathbf{k}}G_{-p}\mathbf{B}\cdot\boldsymbol{g}_{-\mathbf{k}}G_{-p} + \Delta G_p\mathbf{B}\cdot\boldsymbol{g}_{\mathbf{k}}G_p\Delta^\dagger G_{-p}\mathbf{B}\cdot\boldsymbol{g}_{-\mathbf{k}}G_{-p}\right. \\ &\qquad \left. +\Delta G_p\mathbf{B}\cdot\boldsymbol{g}_{\mathbf{k}}G_p\mathbf{B}\cdot\boldsymbol{g}_{\mathbf{k}}G_p\Delta^\dagger G_{-p}\right) \\ &= -2\frac{\mu_B^2(\boldsymbol{d}\cdot\boldsymbol{d}^*)}{\beta}\sum_{p=(\omega_n,\mathbf{k})}\sum_{\tau=\pm}\left[(\mathbf{B}\cdot\boldsymbol{g}_{\tau,-\mathbf{k}})^2G_pG_{-p}^3 + (\mathbf{B}\cdot\boldsymbol{g}_{\tau,\mathbf{k}})(\mathbf{B}\cdot\boldsymbol{g}_{-\tau,-\mathbf{k}})G_p^2G_{-p}^2 + (\mathbf{B}\cdot\boldsymbol{g}_{\tau,\mathbf{k}})^2G_p^3G_{-p}\right] \\ &= -2\frac{\mu_B^2(\boldsymbol{d}\cdot\boldsymbol{d}^*)}{\beta}\sum_{p=(\omega_n,\mathbf{k})}\sum_{\tau=\pm}(\mathbf{B}\cdot\boldsymbol{g}_{\tau,\mathbf{k}})^2\left[G_pG_{-p}^3 - G_p^2G_{-p}^2 + G_p^3G_{-p}\right] \\ &= -\mu_B^2(\boldsymbol{d}\cdot\boldsymbol{d}^*)(N''(0)\log\Lambda\beta - \beta^2N(0))\int_{\mathrm{FS}}d\mathbf{k}\sum_{\tau=\pm}(\mathbf{B}\cdot\boldsymbol{g}_{\tau,\mathbf{k}})^2. \end{aligned} \tag{95}$$

Here, we used $\boldsymbol{g}_{\sigma,\mathbf{k}} = -\boldsymbol{g}_{-\sigma,-\mathbf{k}}$ to go from the second to the third line and (Eq. 92) and (Eq. 93) to go from the third to the fourth line.

Finally, we evaluate the quartic term $(\Delta^\dagger\Delta)^2$ as

$$F_{(\Delta\Delta^\dagger)^2} = \frac{1}{4\beta}\sum_{p=(\omega_n,\mathbf{k})} \mathrm{tr}(\Delta G_p \Delta^\dagger G_{-p})^2 = \frac{1}{2\beta}\mathrm{tr}(\boldsymbol{d}\cdot\boldsymbol{\sigma}\,\boldsymbol{d}^*\cdot\boldsymbol{\sigma})^2 \sum_{p=(\omega_n,\mathbf{k})} G_p^2 G_{-p}^2 \tag{96}$$

The summation over p is given by (93), whereas the trace can be evaluated as

$$\mathrm{tr}(\boldsymbol{d}\cdot\boldsymbol{\sigma}\,\boldsymbol{d}^*\cdot\boldsymbol{\sigma})^2 = \mathrm{tr}(\boldsymbol{d}\cdot\boldsymbol{d}^* + i(\boldsymbol{d}\times\boldsymbol{d}^*)\cdot\boldsymbol{\sigma})^2 = 4(\boldsymbol{d}\cdot\boldsymbol{d}^*)^2 - 2|\boldsymbol{d}\cdot\boldsymbol{d}|^2 \tag{97}$$

leading to

$$F_{(\Delta\Delta^\dagger)^2} = \frac{\beta^2 N(0)}{4}[2(\boldsymbol{d}\cdot\boldsymbol{d}^*)^2 - |\boldsymbol{d}\cdot\boldsymbol{d}|^2] \tag{98}$$

The Free energy now has the form

$$F = \int_{\mathrm{FS}} d\mathbf{k}\left[\boldsymbol{d}\cdot\boldsymbol{d}^*\left(\frac{2}{g} + 2\mu_B^2\beta^2 N(0)(\mathbf{B}\cdot\boldsymbol{g}_{+,\mathbf{k}})^2 - (2N(0) + \mu_B^2 N''(0)(\chi^2\mathbf{B}^2 + 2(\mathbf{B}\cdot\boldsymbol{g}_{+,\mathbf{k}})^2))\log\beta\Lambda\right)\right.$$
$$\left. +4i\mu_B\chi\mathbf{B}\cdot(\boldsymbol{d}\times\boldsymbol{d}^*)N'(0)\log\beta\Lambda + \mu_B^2\chi^2\beta^2 N(0)(\mathbf{B}\cdot\boldsymbol{d})(\mathbf{B}\cdot\boldsymbol{d}^*) + \frac{\beta^2 N(0)}{4}[2(\boldsymbol{d}\cdot\boldsymbol{d}^*)^2 - |\boldsymbol{d}\cdot\boldsymbol{d}|^2]\right]. \tag{99}$$

We notice that the second derivative of the density of states can be estimated as $1/\epsilon_F^2$ which is much smaller that β^2, thus we can throw away all terms containing $N''(0)$. Expanding in T close to $T_c = \Lambda e^{-\frac{1}{gN(0)}}$, we get

$$F = \frac{2N(0)}{T_c}\left[\boldsymbol{d}\cdot\boldsymbol{d}^*\left(T - T_c + \frac{1}{T_c}\int_{\mathrm{FS}} d\mathbf{k}(\mu_B\mathbf{B}\cdot\boldsymbol{g}_{+,\mathbf{k}})^2\right) + 2i\mu_B\mathbf{B}\cdot(\boldsymbol{d}\times\boldsymbol{d}^*)\chi T_c\frac{N'(0)}{N(0)}\log\frac{\Lambda}{T_c}\right.$$
$$\left. + \mu_B^2\chi^2\frac{1}{2T_c}|\mu_B\mathbf{B}\cdot\boldsymbol{d}|^2 + \frac{1}{8T_c}[2(\boldsymbol{d}\cdot\boldsymbol{d}^*)^2 - |\boldsymbol{d}\cdot\boldsymbol{d}|^2]. \right. \tag{100}$$

Comparing with Eq. 6 in the main text, we find that the coefficients $\kappa, a, b, c, \alpha, \eta$ are given by

$$\kappa = \frac{2N(0)}{T_c}, \qquad a = 2\chi T_c\frac{N'(0)}{N(0)}\ln\frac{\Lambda}{T_c}, \qquad b = \frac{1}{T_c}\int_{\mathrm{FS}} d\mathbf{k}(\boldsymbol{e}_{\mathbf{B}}\cdot\boldsymbol{g}_{+,\mathbf{k}})^2, \qquad c = \frac{\chi^2}{2T_c}, \qquad \alpha = -2\eta = \frac{1}{4T_c} \tag{101}$$

where $\boldsymbol{e}_{\mathbf{B}}$ is the direction of the external magnetic field. We notice that the term a was obtained in the description of the superfluid transition in He^3 [15].

[1] Slater, J. C. & Koster, G. F. Simplified lcao method for the periodic potential problem. *Phys. Rev.* **94**, 1498–1524 (1954).
[2] McCann, E. & Koshino, M. The electronic properties of bilayer graphene. *Reports on Progress in Physics* **76**, 056503 (2013).
[3] Charlier, J.-C., Gonze, X. & Michenaud, J.-P. First-principles study of the electronic properties of graphite. *Phys. Rev. B* **43**, 4579–4589 (1991).
[4] Bistritzer, R. & MacDonald, A. H. Moiré bands in twisted double-layer graphene. *Proceedings of the National Academy of Sciences* **108**, 12233–12237 (2011).
[5] Po, H. C., Zou, L., Vishwanath, A. & Senthil, T. Origin of mott insulating behavior and superconductivity in twisted bilayer graphene. *Phys. Rev. X* **8**, 031089 (2018).
[6] Moon, P. & Koshino, M. Optical absorption in twisted bilayer graphene. *Phys. Rev. B* **87**, 205404 (2013).
[7] Koshino, M. *et al.* Maximally localized wannier orbitals and the extended hubbard model for twisted bilayer graphene. *Phys. Rev. X* **8**, 031087 (2018).
[8] Kohmoto, M. Topological invariant and the quantization of the hall conductance. *Annals of Physics* **160**, 343 – 354 (1985).
[9] Zhang, Y.-H., Mao, D., Cao, Y., Jarillo-Herrero, P. & Senthil, T. Nearly flat chern bands in moiré superlattices. *Phys. Rev. B* **99**, 075127 (2019).
[10] Xie, M. & MacDonald, A. H. On the nature of the correlated insulator states in twisted bilayer graphene. *arXiv preprint arXiv:1812.04213* (2018).
[11] Jung, J. & MacDonald, A. H. Accurate tight-binding models for the π bands of bilayer graphene. *Phys. Rev. B* **89**, 035405 (2014).
[12] Carr, S., Fang, S., Zhu, Z. & Kaxiras, E. Minimal model for low-energy electronic states of twisted bilayer graphene. *arXiv e-prints* arXiv:1901.03420 (2019).

[13] Bultinck, N., Chatterjee, S. & Zaletel, M. P. Anomalous hall ferromagnetism in twisted bilayer graphene. *arXiv preprint arXiv:1901.08110* (2019).
[14] Usov, N. Theory of the quantum hall effect in a two-dimensional periodic potential. *JETP* **67**, 2565 (1988).
[15] Ambegaokar, V. & Mermin, N. D. Thermal anomalies of he^3: Pairing in a magnetic field. *Phys. Rev. Lett.* **30**, 81–84 (1973).
